# Knowledge mapping of DNGR-1 on dendritic cells in immunotherapy: a review and bibliometric study

**DOI:** 10.3389/fimmu.2026.1748597

**Published:** 2026-05-01

**Authors:** Fang Liu, Xingkai Yang, Yixuan Sun, Wei Zhao, Zhongyi Yan

**Affiliations:** Lab of Nano-Immunotherapy, School of Basic Medical Sciences, Henan University, Kaifeng, China

**Keywords:** bibliometrics, CLEC9A, dendritic cells, DNGR-1, tumor immunotherapy, vaccines

## Abstract

**Introduction:**

DNGR-1 (CLEC9A), a C-type lectin receptor specifically expressed on human BDCA3+ and murine CD8α+/CD103+ conventional type 1 dendritic cells (cDC1), acts as a damage-associated molecular pattern (DAMP) receptor that recognizes necrotic cell-derived F-actin and promotes antigen cross-presentation. Despite the growing focus on DNGR-1 in immunotherapy research, no comprehensive, global bibliometric analysis spanning the full research history of DNGR-1 has been reported to date to delineate its global research trends, hotspots, and evolutionary trajectories.

**Methods:**

We retrieved 326 relevant DNGR-1 publications from the Web of Science Core Collection (WOSCC) and Scopus databases, covering the period from January 2008 to January 2026. Bibliometric visualization and analysis were performed using VOSviewer, CiteSpace, and Bibliometrix, with associated clinical trial data retrieved from PubMed to contextualize translational progress.

**Results:**

Annual publication output in this field has shown a steady upward trend, with the USA, the UK, and China ranking as the top three contributing countries. Monash University was the most productive institution (32 papers), Lahoud MH was the most prolific author (33 papers), and Sancho D was the most frequently co-cited researcher (228 citations). Frontiers in Immunology was the most prolific journal in this field. Thematic evolution in the field has shifted from an early focus on "C-type Lectins" and "myeloid cells" to recent research centered on "Adaptive Immunity", "Spatial Transcriptomics", and "Systems Biology", reflecting a clear evolution from foundational molecular mechanism research to cross-scale translational immunotherapy development.

**Conclusion:**

This study presents the first systematic bibliometric analysis of global DNGR-1 research, mapping the knowledge structure, research hotspots, and evolutionary trends of the field over the 18-year study window. Our integrated analysis of publication trends, clinical trial data, and research frontiers highlights the critical role of DNGR-1 in tumor immunotherapy and cDC1 functional regulation, providing theoretical and empirical insights for developing novel immunotherapies targeting the DNGR-1-cDC1 axis.

## Introduction

Dendritic cells (DC) are antigen-presenting cells that orchestrate and regulate innate and adaptive immunity (1, 2). DC are typically divided into three subgroups, BATF3 dependent cDC1 (CD8α^+^ and CD103^+^ DC), IRF4 dependent cDC2 (CD11b^+^ and CD172a^+^ DC) and E2–2 dependent plasmacytoid DC (pDC) (3). cDC1 and cDC2 regulate T helper cell 1(Th1) and Th2-type immune responses, respectively. pDC is specialized producers of Type I interferon (IFN-I), which can promote anti-viral and anti-tumor immunity (4). Notably, cDC1 can cross-present and load cell-exogenous antigens as peptides onto MHC-I molecules to CD8^+^ T cells, thereby exerting immunity against tumors and viruses (5). Importantly, the cross-presentation functions are partly due to the unique membrane biomarkers proposed for cDC1 (3). With the development of high-dimensional flow cytometry and single-cell transcriptomics, DC subsets can be further divided into DC3 and monocyte-derived DC (moDC). Murine DC3 (CD16/32^+^CD172a^+^) have a distinct developmental origin from monocytes and other DC subsets, differentiating from Ly6C^+^ monocyte-DC progenitors through Lyz2^+^Ly6C^+^CD11c^-^ pro-DC3, and possess a strong potential to polarize Th17 cells (6, 7); moDC (CD88^+^CD26^-^) originate from monocytes, and upon activation by TLR7 agonists, can differentiate into antigen-cross-presenting APCs to induce antigen-specific CTL responses (8, 9).

In 2008, Huysamen et al. first described CLEC9A (a member of the C-type lectin structural domain family 9), which is also known as DNGR-1 and CD370 (10). This receptor follows standard species-specific nomenclature: it is designated CLEC9A in human research, Clec9a in murine study, and DNGR-1 referred to across species. DNGR-1 contains an extracellular C-type lectin-like structural domain, transmembrane structural domain, and an ITAM-like motif in its cytoplasmic tail (10). DNGR-1 is highly restricted and is expressed by cross-presenting DC subsets in both murine CD8α^+^ and CD103^+^CD11b^-^ cDC1 and human BDCA3^+^ DC (11, 12). As a DC-specific damage-associated molecular pattern receptor, Clec9a recognizes and binds damaged cells via exposed actin filaments and promotes the Clec9a-dependent cross-presentation of dead cell-associated Ag with myosin II (13–18). As a promising strategy to improve DC-targeting vaccine effectiveness, many researches utilize Clec9a antibody or affinity peptides to deliver antigen targeting to Clec9a^+^ DC, which strongly enhances cellular and humoral immune responses (17, 19–23). In addition, Clec9a induces intracellular signaling pathways by recruiting spleen tyrosine kinase (SYK) and enabling endocytic cargo shuttling into the cross-presentation pathway (10, 18, 24). In mouse experiments, deletion or blockade of Clec9a does not affect CD8α^+^ DC uptake of necrotic cellular material (13), but Clec9a as a rheostat can limit tissue damage by dampening neutrophil recruitment (25).

As a mature scientific discipline, bibliometrics uses mathematical and statistical methods to quantitatively analyze and objectively reflect research hotspots and predict future trends (26, 27). DNGR-1 is gradually gaining more attention among scholars. However, there is a lack of systematic review of the evolution of research hotspots in this field, resulting in a cognitive fault between the basic mechanism and clinical transformation. Currently, there are 326 published papers on DNGR-1, with USA leading in publication volume at 79 papers. Lahoud, Mireille H. published 33 articles as the first among authors, and Sancho, D. published 228 co-citations as co-authors. The present study evaluated the literature on DNGR-1 from January 1, 2008, to January 31, 2026 based on a large amount of data to characterize the trends in the field and identify new research directions.

## Materials and methods

### Data collection

**Figure 1:**
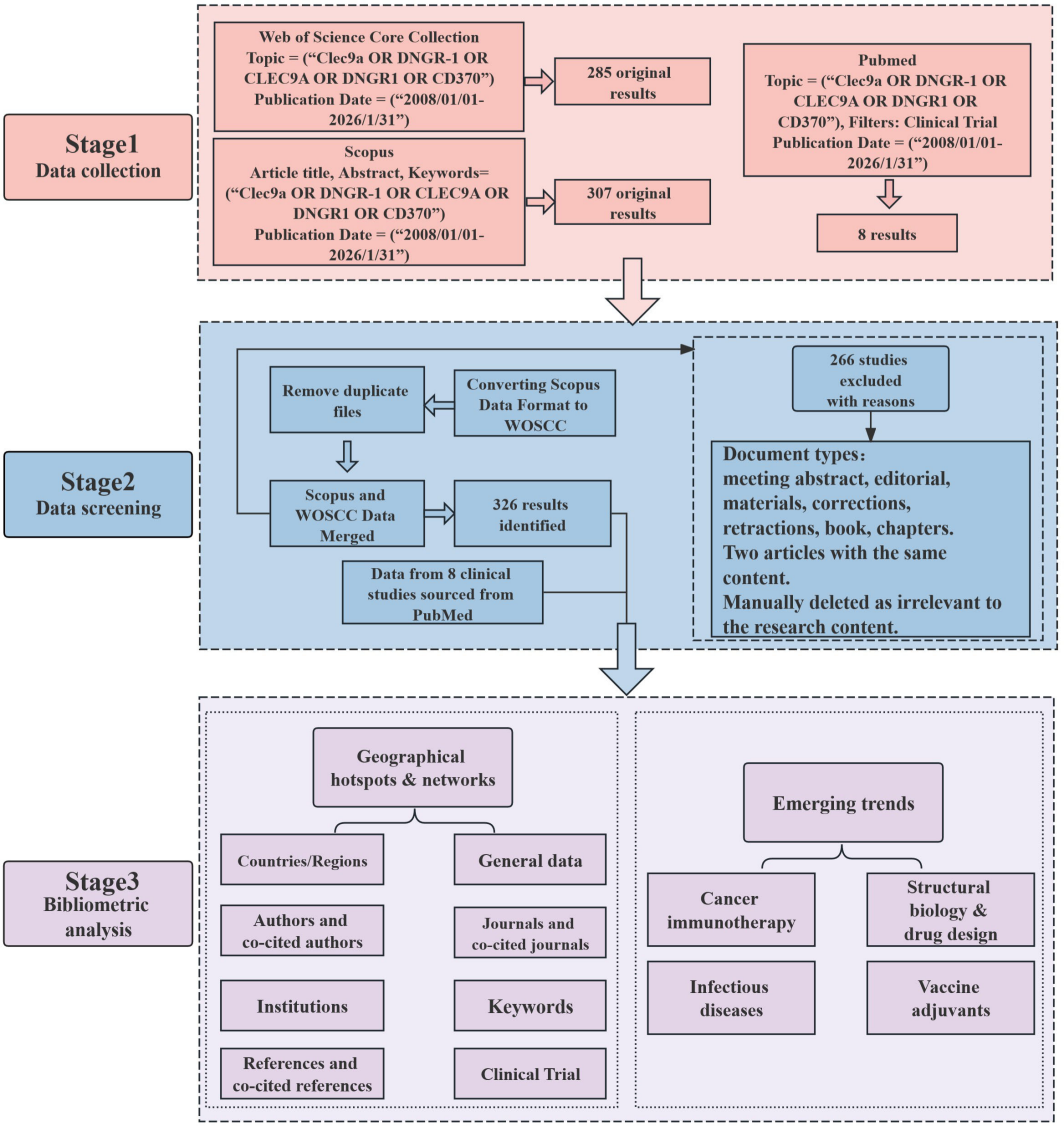
Data collection and bibliometric analysis process.

We retrieved relevant literature on DNGR-1 from the Web of Science Core Collection (WOSCC) and Scopus between 1 January 2008 and 31 January 2026. The Science Citation Index Expanded (SCI-E) served as the data source, with document types restricted to “articles” and “review articles”. Primary search terms were “Clec9a”, “CD370” and “DNGR-1”. The search formula employed was (TS=(Clec9a) OR TS = (DNGR-1) OR TS=(CLEC9A) OR TS = (DNGR1) OR TS = (CD370)). Data collected from both databases were merged and screened, yielding a total of 326 publications (**Figure 1**, **Supplementary Table 1**).

Inclusion criteria: (1) Literature type being article and review article; (2) English-language publications.

Exclusion criteria: (1) Duplicate publications; (2) Literature types including conference abstracts, web-first publications, editorial materials, proceedings, letters to the editor, revised manuscripts, and news reports; (3) Literature not relevant to the research topic.

The following search query was used in PubMed to collect clinical trial data related to CLEC9A: ((Clec9a[Title/Abstract]) OR (DNGR1[Title/Abstract]) OR (CLEC9A[Title/Abstract]) OR (DNGR-1[Title/Abstract]) OR (CD370[Title/Abstract])) AND ((‘2008/1/1’ [Publication Date]: ‘2026/1/31’ [Publication Date])), with the filter criteria: Clinical Trials.

### Statistical methods

Microsoft Office Excel 2019 (Microsoft, Redmond, Washington, USA) was used to process the data and construct graphs of publication trends, with fluctuating, but gradually increasing trends in the number of publications per year. Bibliometrix was used to create the visual maps and Sankey diagrams. The visual maps provide a visual depiction of the national distribution of publications, whereas the Sankey diagrams can be used to depict the relationships between the three domains of authors/keywords/countries. VOSviewer (1.6.18) explored the author/collaborative networks between institutes/countries/journals. In VOSviewer, nodes are used to represent the analyzed elements, such as countries, institutes, journals, and authors, where different colors indicate different clusters, their size depends on their co-occurrence frequency in titles and abstracts, and connections between nodes represent collaborative and co-occurring relationships (28). CiteSpace 6.1.R6 (Joyce M. Chen, Drexel University, USA) extracted keywords and references from highly cited bursts of publications and constructed a bimap overlay of journals. Thus, CiteSpace was used to investigate research trends on a given topic (29). The CiteSpace parameters included the following: link retention factor (LRF = 3), e for top N (e = 1), Maximum Links Per Node (L/N = 10) time span (2008-2026), years per slice (1), year of review (LBY = 5), links (strength: cosine, scope: within slices), selection criteria (g-index: k = 25), and minimum duration (MD = 1).

## Results

### DNGR-1 research milestones: a chronological perspective (2008-2026)

**Figure 2:**
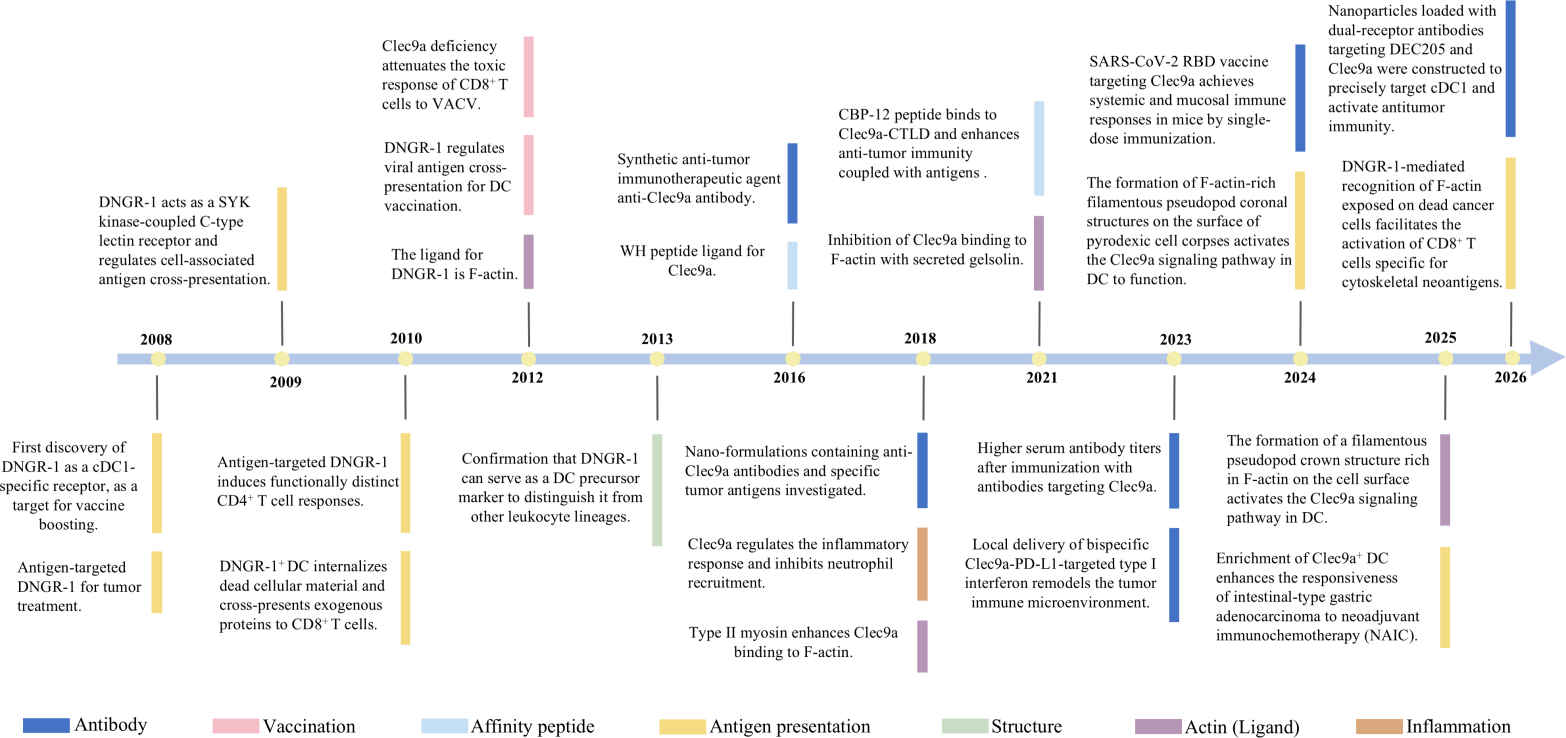
Time line of DNGR-1 research.

DNGR-1, a crucial molecule expressed on DC, has garnered significant research attention in recent years (**Figure 2**), prompting extensive investigation of its functional mechanisms and therapeutic potential. (1) Discovery and functional elucidation (2008-2010). First identify of DNGR-1 as a cDC1-specific receptor for antigen uptake that senses dead cells (10–12). Mechanistically, it has demonstrated its capacity to recognize F-actin exposed by necrotic cells via a SYK-dependent signaling pathway, establishing its role in dead cell surveillance (12, 13). Defined DNGR-1 essential role in the cross-presentation of cell-associated antigens through adoptive transfer experiments. The conceptual framework of “corpse-clearing receptors” in immune homeostasis was validated by impaired CD8^+^ T-cell priming in Clec9a^-/-^ mice (30, 31). (2) Mechanistic and structural revelations (2012-2015). The ligand of DNGR-1 is F-actin (14, 16, 32). The crystal structure of DNGR-1 extracellular domain, revealing a distinctive trefoil conformation with exposed amino acid residues (Trp155, Trp250, and Lys251) that were critical for F-actin binding. In addition, DNGR-1 as a lineage tracing biomarker of DC can distinct hematopoietic cell types (33). (3) Antigen-targeting strategies (2016-2021). Since its discovery, researchers have investigated the DNGR-1 functional roles, particularly in immune responses. This phase is marked by studies that have elucidated its involvement in DC biology and antigen presentation. As promising target, Clec9a directed antibody-antigen conjugates have demonstrated the ability to elicit robust antitumor immune responses by selectively engaging cDC1 (24, 34–43). Yan et al. discovered that the WH and CBP-12 peptides coupled with antigens bind to Clec9a-CTLD to enhance antitumor immunity (21, 44). As a nanocarrier, the Clec9a-TNE nanoemulsion enables adjuvant-free antigen delivery and elicits antigen-specific immunotherapy (37, 45, 46). In anti-viral immunity, Clec9a deficient mice mount reduced CD8^+^ T cell responses against viral diseases, such as herpes simplex virus-1 (24). These conjugates leverage Clec9a unique role in recognizing damage-associated molecular patterns (e.g., F-actin) and routing antigens to MHC-I cross-presentation pathways, thereby enhancing CTL activation while minimizing off-target effects. This strategy capitalizes on Clec9a specificity for cDC1-a subset critical for antitumor immunity, making it a promising platform for next-generation cancer vaccines. (4) Clinical translation advancements (2022-). With the accumulating knowledge of its biological roles and disease associations, researchers have started exploring the clinical applications of DNGR-1 (47). These biomarkers include potential diagnostic biomarkers and therapeutic targets. By targeting type I interferon, a bispecific antibody (Clec9a×PD-L1) enabled the spatial-temporal coordination of DC activation and T-cell reinvigoration towards an antitumor state (48). The SARS-CoV-2 RBD vaccine targeting Clec9a achieved systemic and mucosal immune responses in mice via single-dose immunization (49). The formation of F-actin-rich filamentous pseudopod coronal structures on the surface of cell corpses activates the Clec9a signaling pathway in DC to function (50). The latest phase involves emerging technologies that could enhance our understanding of Clec9a, such as CRISPR/Cas9 gene editing techniques. This opens new avenues for research and therapeutic applications.

### Annual growth trend of publication

**Figure 3:**
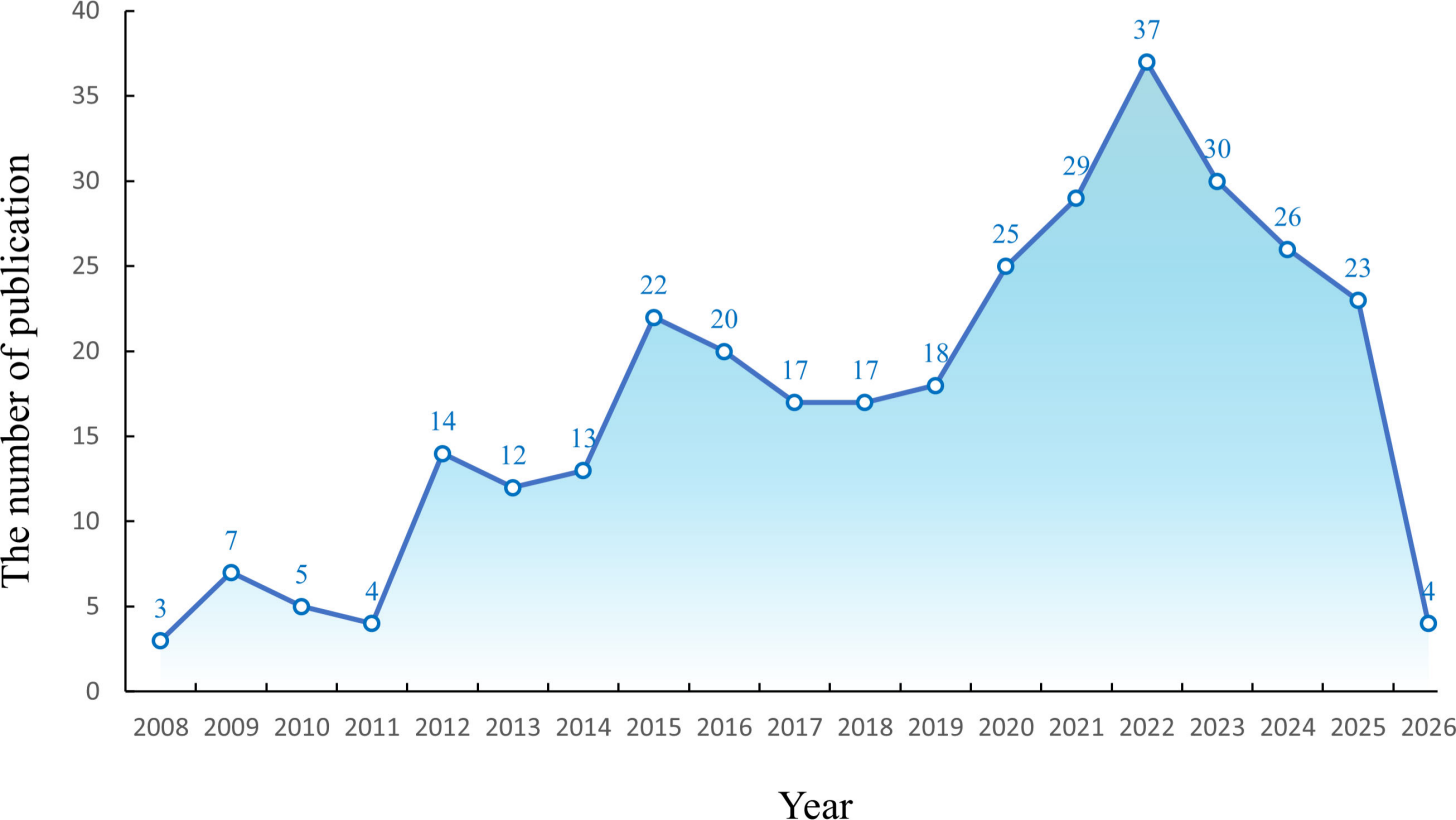
Annual publication volume graph.

After a comprehensive search of the WOSCC (Web of Science Core Collection) and Scopus database, 326 documents, consisting of 277 research articles and 49 review articles related to DNGR-1 research from 2008 to 2026, were retrieved. The annual number of publications exhibits a zigzag growth pattern from 2008 to 2026(**Figure 3**). The highest number of publications occurred in 2022, with 37 items, accounting for 11.35% of the total. By contrast, the lowest number of articles was published in 2008, with only three articles representing 0.92% of the total. Overall, although the annual number of publications fluctuated, a gradual upward trend was observed.

### A bibliometric perspective of global research productivity and collaborative dynamics in scholarly output

**Figure 4:**
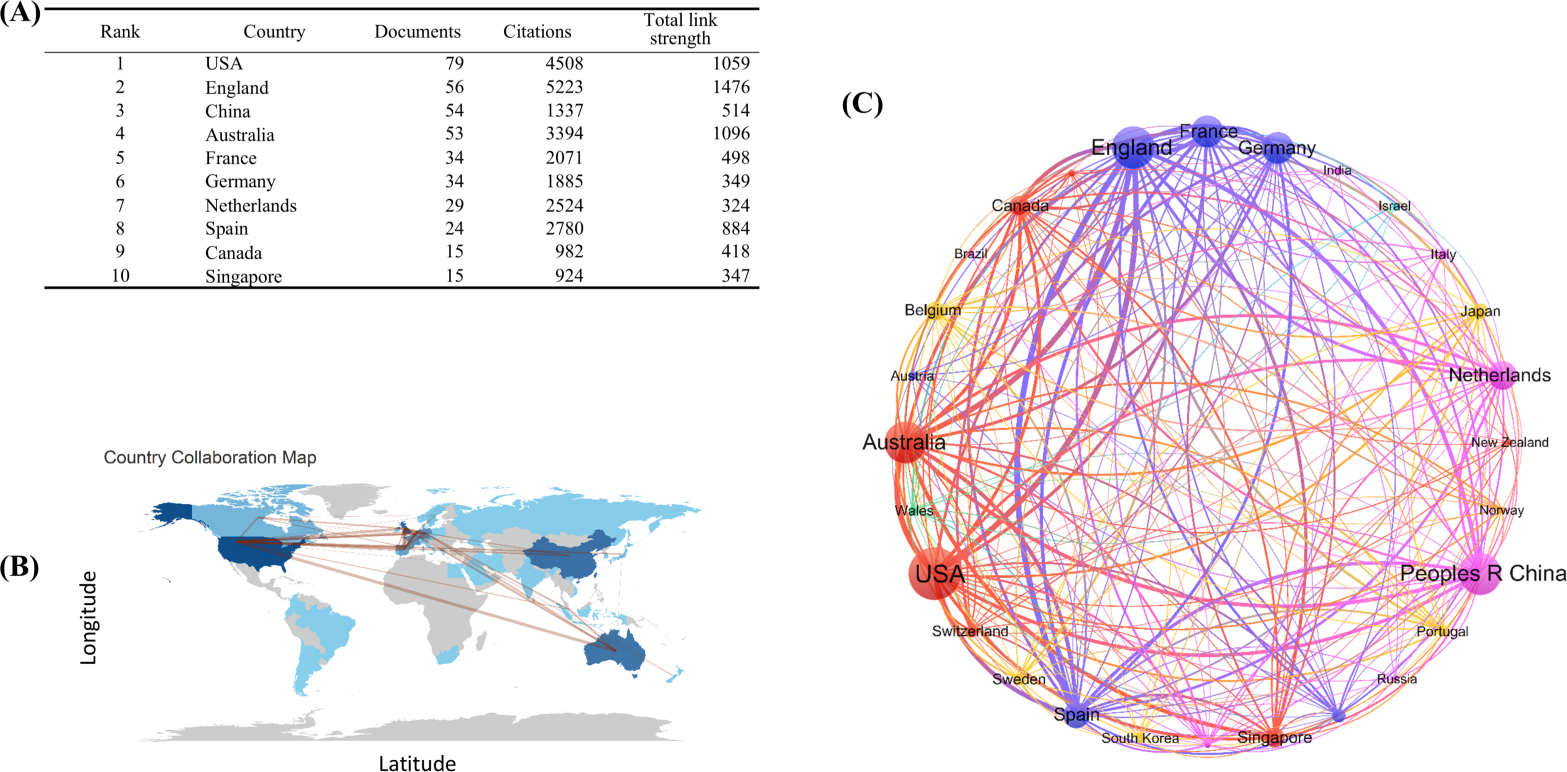
Analysis of countries/regions participating in DNGR-1-related studies. **(A)** Top 10 most efficient countries/regions. **(B)** Distribution of countries in terms of publications. **(C)** The visualization map of international collaboration countries. Each node represents a country/region.

A comprehensive analysis of 326 academic articles spanning 42 countries/regions has revealed distinct geographical productivity patterns and intricate cooperative networks. The United States (USA) emerged as the predominant contributor with 79 publications, closely trailed by England (UK) 56 articles and China 54 outputs. However, the UK exhibited the highest collaborative connectivity with other nations, boasting a total link strength of 1,476, surpassed only by Australia (total link strength = 1,096) and USA (total link strength = 1,059) (**Figures 4A, B**). International collaboration patterns revealed significant partnerships between Australia, China, UK, and USA, with cross-border co-operation density notably exceeding that of single-country contributions (**Figure 4C**).

**Figure 5:**
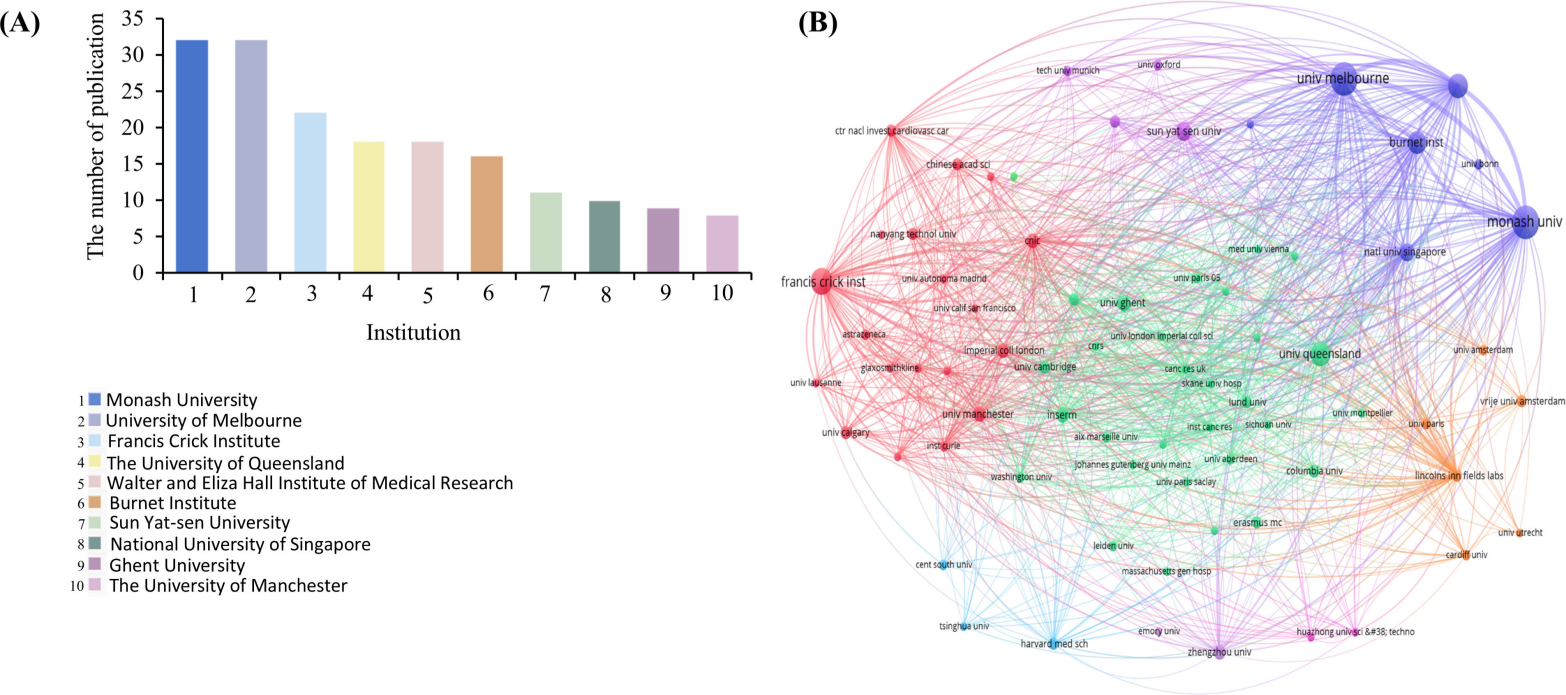
Analysis of institutions involved in DNGR-1-related research. **(A)** Top 10 institutions involved in DNGR-1-related research. **(B)** Network diagram of institutions participating in DNGR-1-related research.

The analysis encompassed contributions from 596 organizations, with the top 10 institutions accounting for 176 articles (54.0% of the total publications, **Figure 5A**). Notably, Australian institutions dominated the ranking by occupying five positions in the Top10. The University of Melbourne and Monash University were the top-producing institutions (n=32, 9.8%). Institutional collaboration networks (**Figure 5B**) exhibited greater complexity than international partnerships, particularly exemplified by Monash University’s extensive partnerships with multiple Chinese academic institutions and research centers, alongside collaborations with Singaporeans, Americans, and other international organizations.

### Authors and co-cited authors

**Figure 6:**
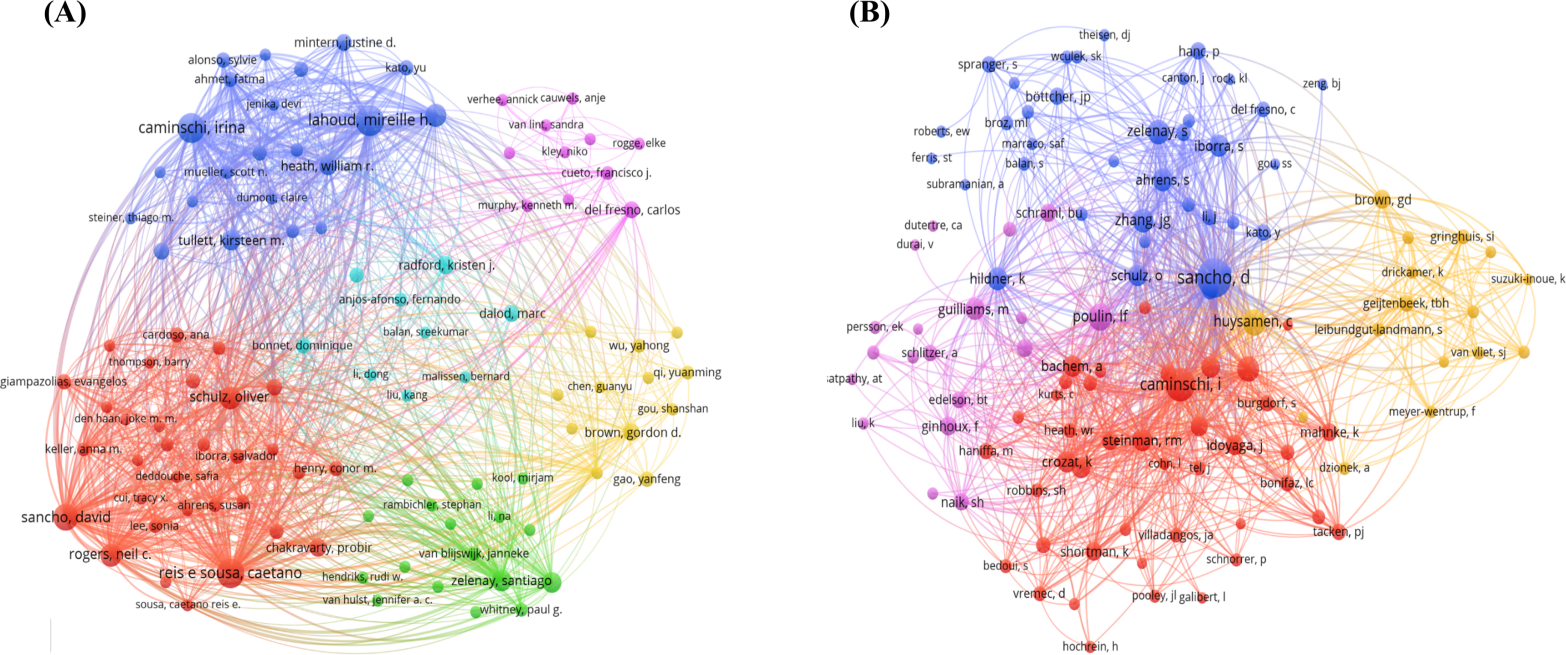
A network diagram of authors **(A)** or co-cited authors **(B)** involved in the DNGR-1 study.

The bibliometric analysis of DNGR-1-related research involved more than 2,000 investigators, with Lahoud et al. (n = 33), Reis e Sousa, Caetano (n = 33), and Caminschi, Irina (n = 30) identified as the most prolific contributors (**Table 1**). Co-citation analysis revealed that Sancho (228 citations), Caminschi, Irina (159), and Poulin Lionel (98) were the predominant intellectual influencers. Network visualization via VOSviewer demonstrated two distinct patterns: 1) author collaboration clusters, where node diameter correlated with publication volume, and 2) co-citation linkages exhibiting a core-periphery structure (**Figures 6A, B**).

**Table 1:** Top 10 authors and co-citing authors involved in DNGR-1-related studies.

Rank	Author	Counts	Co-cited author	Counts
1	Lahoud, Mireille H	33	Sancho David	228
2	Reis e sousa, Caetano	33	Caminschi, Irina	159
3	Caminschi, Irina	30	Poulin Lionel F	98
4	Sancho, David	22	Huysamen Cristal	95
5	Shortman, Ken	15	Lahoud, Mireille H	85
6	Rogers, Neil C	14	Zelenay Santiago	83
7	Schulz, Oliver	14	Zhang Jian-guo	76
8	Tullett, Kirsteen M	13	Hildner Kai	72
9	Zelenay, Santiago	12	Ahrens Susan	70
10	Heath, William R	11	Salvador Iborra	67

### Journals and co-cited academic journals

**Figure 7:**
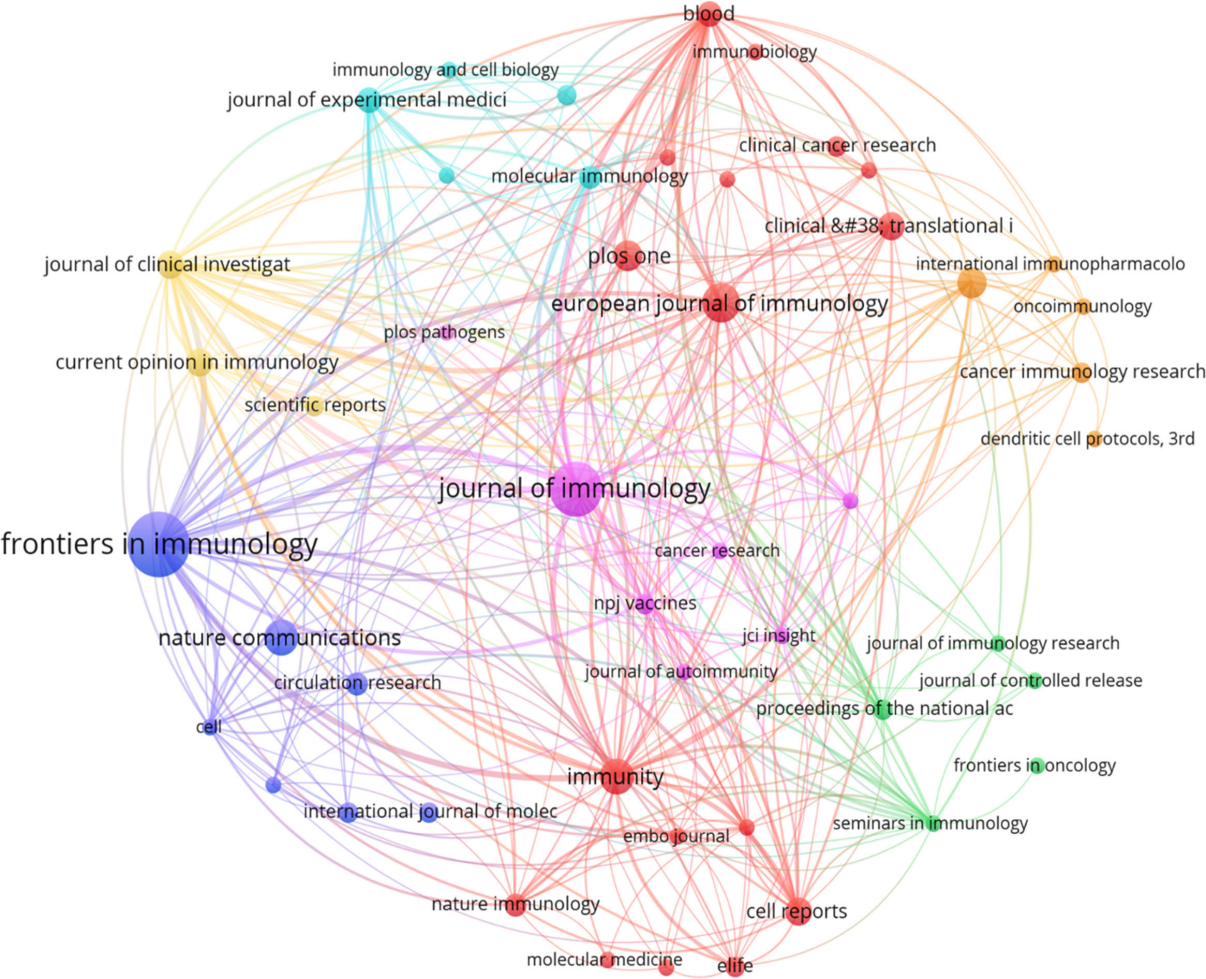
Network map of academic journals associated with DNGR-1 research.

A total of 256 academic journals published articles related to DNGR-1, with Frontiers in Immunology (n = 29, IF2025 = 5.9) ranking first in Switzerland, followed by the Journal of Immunology (n =21, IF2025 = 3.4). Among the top 10 journals (**Table 2**), 60% (6/10) were from USA, followed by 30% (3/10) from the UK. The highest impact factor among the top ten journals was Immunity (n = 9, IF2025 = 26.3). As shown in **Figure 7**, there is a positive citation relationship between the different journals.

**Table 2:** The top 10 academic journals involving research related to DNGR-1.

Rank	Journal	Counts	Percent	Country	IF (2025)
1	Frontiers in Immunology	29	8.90%	Switzerland	5.9
2	Journal of Immunology	21	6.44%	USA	3.4
3	European Journal of Immunology	11	3.37%	USA	3.7
4	Immunity	9	2.76%	USA	26.3
5	Nature Communications	9	2.76%	UK	15.7
6	PLOS One	7	2.15%	USA	2.6
7	Journal for Immunotherapy of Cancer	7	2.15%	UK	10.6
8	Journal of Clinical Investigation	6	1.84%	USA	13.6
9	Cell Reports	6	1.84%	USA	6.9
10	Current Opinion in Immunology	6	1.84%	UK	5.8

The publication landscape analysis revealed distinct contributions from premier journals: One-related to DNGR-1 article was published in Nature, three studies in Cell, and two papers in Science (**Table 3**). Among CNS-affiliated journals, nature-branded titles dominated with 19 publications (55.88% of the total CNS sub-journal output), followed by cell-affiliated platforms (n=10, 29.41%).

**Table 3:** CNS journals and their sub-journals involved in research related to DNGR-1.

Journal	Counts	IF (2025)
Cell	3	42.5
Cell Reports	7	6.9
Nature	1	48.5
Nature Communications	10	15.7
Nature Immunology	4	27.6
Nature Reviews Immunology	2	60.9
Nature Cell Biology	1	19.1
Nature Cancer	1	28.5
Science	2	45.8
Science Advances	2	12.5
Science Signaling	1	6.6

**Figure 8:**
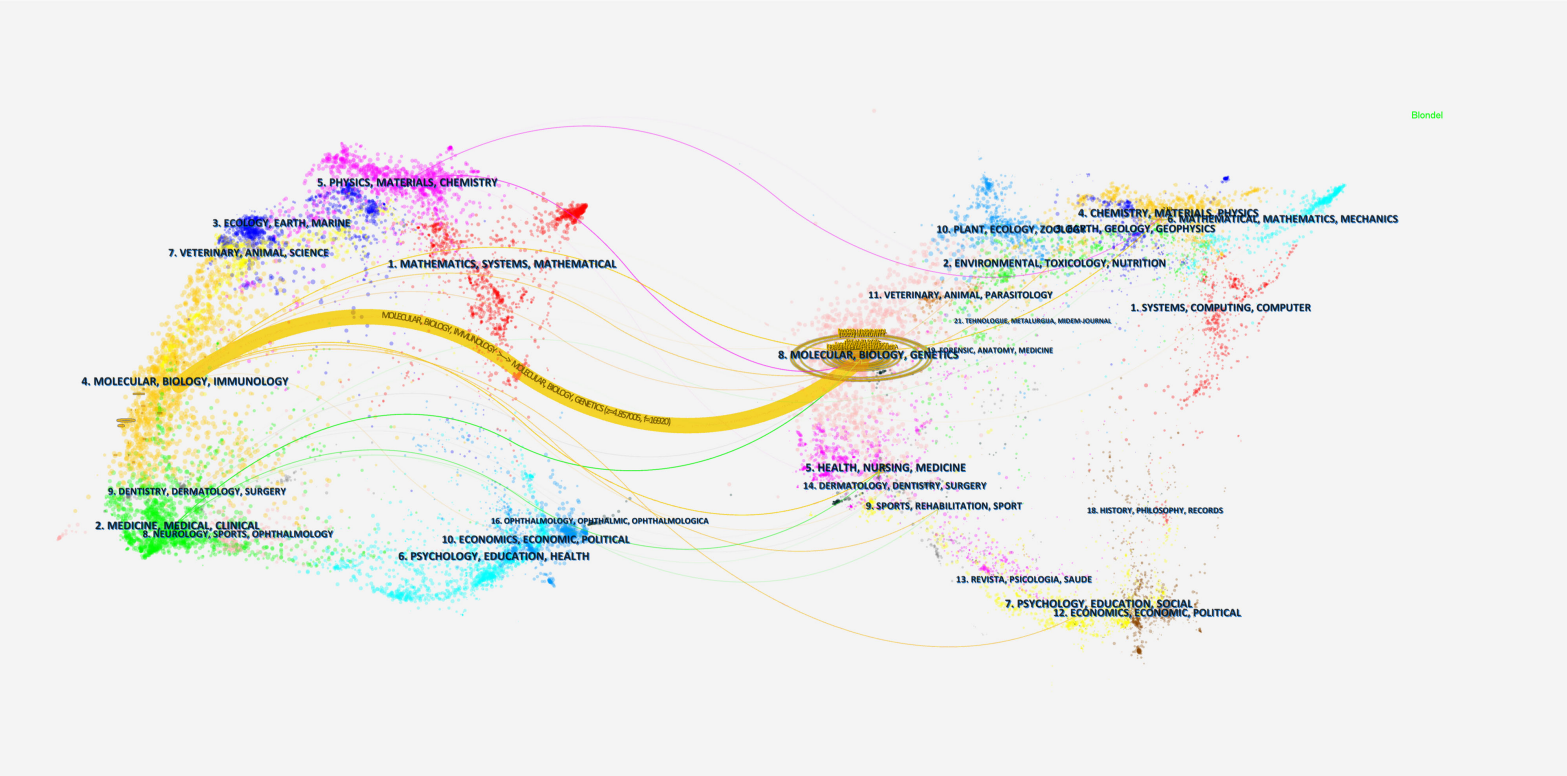
Dual graph overlay analysis of journals associated with the DNGR-1 study.

The double-map overlay analysis delineates interdisciplinary citation patterns at the journal level (**Figure 8**). This visualization technique effectively maps knowledge transfer between disciplines through journal citations, where the left hemisphere represents cited journals (knowledge sources) and the right hemisphere denotes citing journals (knowledge recipients). Key observations revealed a distinctive citation corridor: molecular/biology/immunology journals (source domain) demonstrated significant knowledge export to molecular/biology/genetics journals (recipient domain), as evidenced by a singular yellow citation trajectory with remarkable statistical parameters (z-score = 4.857, f = 16,920). This pathway not only indicates substantial disciplinary interaction but also reflects the evolutionary trajectory of life science research, where immunological discoveries increasingly inform genetic investigations.

Notably, a z-score exceeding 3.0 threshold confirms citation patterns beyond random expectation (*p* < 0.01), while the frequency count (f-value) quantifies the absolute citation volume between these domains. Such directional citation flows suggest potential paradigm shifts, in which genetic research systematically builds upon immunological frameworks through cumulative citation behaviors.

### Analysis of co-cited references

As delineated in **Table 4**, enumerating the ten most frequently cited references, the seminal study by Caminschi et al. (11) entitled “The dendritic cell subtype-restricted C-type lectin Clec9a is a target for vaccine enhancement” emerged as the most influential work with 108 citations. This landmark publication in Blood (IF2008 = 10.4) demonstrated Clec9a’s pivotal role as a DC-specific surface receptor that enhances antigen cross-presentation, thus establishing it as a promising target for vaccine adjuvants. Mechanistic insights into cDC1-specific targeting strategies have profoundly shaped modern vaccine development.

**Table 4:** The top 10 co-citations of the DNGR-1 study.

Rank	Co-cited reference	Counts	Strength	IF(2025)
1	11, Blood, V112, P3264	108	1023	23.1
2	12, J Clin Invest, V118, P2098	104	994	13.6
3	13, Nature, V458, P899	101	951	48.5
4	10, J Biol Chem, V283, P16693	89	822	3.9
5	16, Immunity, V36, P646	75	759	26.3
6	51, Science, V322, P1097	72	657	45.8
7	14, Immunity, V36, P635	69	685	26.3
8	17, 2012, J Clin Invest, V122, P1615	67	638	13.6
9	52, J Exp Med, V207, P1261	54	567	10.6
10	39, J Immunol, V187, P842	54	533	3.4

**Figure 9:**
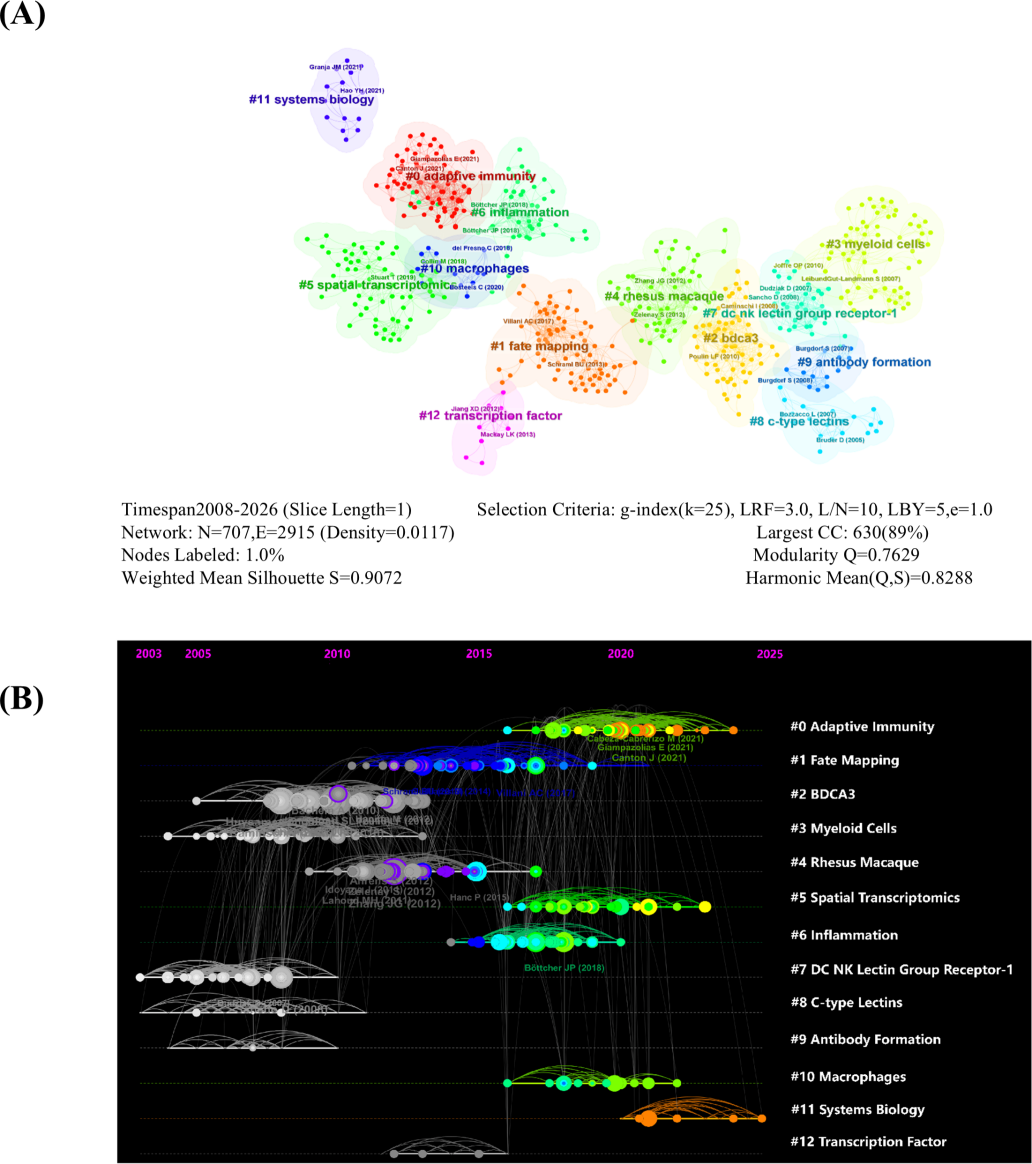
Knowledge graph **(A)** and timeline view **(B)** of references related to the DNGR-1 study.

The co-citation network of references, constructed using CiteSpace, identified 13 clusters (**Figure 9A**) with high modularity (Q = 0.7629, >0.3) and mean silhouette score (Q = 0.9072, >0.7), confirming robust clustering. The two largest clusters, labeled #0 (Adaptive Immunity) and #1 (Fate Mapping), dominate the knowledge graph. A timeline visualization (**Figure 9B**) revealed chronological research trends, where node colors indicate publication years (left: older; right: newer), and clusters such as #2 (BDCA3), #0 (Adaptive Immunity), and #5 (Spatial Transcriptomics) highlight evolving interdisciplinary connections. Citation burst analysis identified 25 important references (**Table 5**), with recent highlights including Böttcher et al. (59, Cell; burst: 2020–2023, strength = 6.67) (1), Giampazolias et al. (2021, Cell; burst: 2022–2026, strength = 6.04) (61), and Canton et al. (60, Nat Immunol; burst: 2022–2026, strength = 7.92) (60), underscoring the advances in vaccine design and innate immunity mechanisms.

**Table 5:** The top 25 references with the strongest citation bursts.

References	Year	Strength	Begin	End	2008-2026
11, BLOOD, V112, P3264, DOI 10.1182/blood-2008-05-155176	2008	13.42	2009	2013	▂▃▃▃▃▃▂▂▂▂▂▂▂▂▂▂▂▂▂
12, J CLIN INVEST, V118, P2098, DOI 10.1172/JCI34584	2008	12.57	2009	2013	▂▃▃▃▃▃▂▂▂▂▂▂▂▂▂▂▂▂▂
10, J BIOL CHEM, V283, P16693, DOI 10.1074/jbc.M709923200	2008	11.72	2009	2013	▂▃▃▃▃▃▂▂▂▂▂▂▂▂▂▂▂▂▂
13, NATURE, V458, P899, DOI 10.1038/nature07750	2009	10.22	2010	2014	▂▂▃▃▃▃▃▂▂▂▂▂▂▂▂▂▂▂▂
53, J EXP MED, V207, P1273, DOI 10.1084/jem.20100348	2010	8.5	2011	2015	▂▂▂▃▃▃▃▃▂▂▂▂▂▂▂▂▂▂▂
54, J EXP MED, V207, P1283, DOI 10.1084/jem.20100223	2010	7.71	2011	2015	▂▂▂▃▃▃▃▃▂▂▂▂▂▂▂▂▂▂▂
52, J EXP MED, V207, P1261, DOI 10.1084/jem.20092618	2010	11.99	2012	2015	▂▂▂▂▃▃▃▃▂▂▂▂▂▂▂▂▂▂▂
30, J EXP MED, V207, P1247, DOI 10.1084/jem.20092140	2010	10.02	2012	2015	▂▂▂▂▃▃▃▃▂▂▂▂▂▂▂▂▂▂▂
39, J IMMUNOL, V187, P842, DOI 10.4049/jimmunol.1101176	2011	6.73	2012	2016	▂▂▂▂▃▃▃▃▃▂▂▂▂▂▂▂▂▂▂
16, IMMUNITY, V36, P646, DOI 10.1016/j.immuni.2012.03.009	2012	11.63	2013	2017	▂▂▂▂▂▃▃▃▃▃▂▂▂▂▂▂▂▂▂
14, IMMUNITY, V36, P635, DOI 10.1016/j.immuni.2012.03.008	2012	8.96	2013	2017	▂▂▂▂▂▃▃▃▃▃▂▂▂▂▂▂▂▂▂
55, IMMUNITY, V37, P60, DOI 10.1016/j.immuni.2012.04.012	2012	8.27	2013	2015	▂▂▂▂▂▃▃▃▂▂▂▂▂▂▂▂▂▂▂
24, J CLIN INVEST, V122, P1628, DOI 10.1172/JCI60660	2012	7.83	2013	2017	▂▂▂▂▂▃▃▃▃▃▂▂▂▂▂▂▂▂▂
15, BLOOD, V119, P6052, DOI 10.1182/blood-2012-01-406967	2012	6.7	2013	2017	▂▂▂▂▂▃▃▃▃▃▂▂▂▂▂▂▂▂▂
17, J CLIN INVEST, V122, P1615, DOI 10.1172/JCI60644	2012	9.55	2014	2017	▂▂▂▂▂▂▃▃▃▃▂▂▂▂▂▂▂▂▂
33, CELL, V154, P843, DOI 10.1016/j.cell.2013.07.014	2013	5.98	2015	2018	▂▂▂▂▂▂▂▃▃▃▃▂▂▂▂▂▂▂▂
3, NAT REV IMMUNOL, V14, P571, DOI 10.1038/nri3712	2014	6.64	2016	2019	▂▂▂▂▂▂▂▂▃▃▃▃▂▂▂▂▂▂▂
32, IMMUNITY, V42, P839, DOI 10.1016/j.immuni.2015.04.009	2015	5.81	2016	2020	▂▂▂▂▂▂▂▂▃▃▃▃▃▂▂▂▂▂▂
56, SCIENCE, V356, P0, DOI 10.1126/science.aah4573	2017	7.19	2018	2022	▂▂▂▂▂▂▂▂▂▂▃▃▃▃▃▂▂▂▂
25, SCIENCE, V362, P351, DOI 10.1126/science.aan8423	2018	5.94	2019	2021	▂▂▂▂▂▂▂▂▂▂▂▃▃▃▂▂▂▂▂
59, CELL, V172, P1022, DOI 10.1016/j.cell.2018.01.004	2018	6.67	2020	2023	▂▂▂▂▂▂▂▂▂▂▂▂▃▃▃▃▂▂▂
57, CANCER CELL, V31, P711, DOI 10.1016/j.ccell.2017.04.003	2017	5.95	2020	2022	▂▂▂▂▂▂▂▂▂▂▂▂▃▃▃▂▂▂▂
60, NAT IMMUNOL, V22, P140, DOI 10.1038/s41590-020-00824-x	2021	7.92	2022	2026	▂▂▂▂▂▂▂▂▂▂▂▂▂▂▃▃▃▃▃
58, ANNU REV IMMUNOL, V39, P131, DOI 10.1146/annurev-immunol-061020-053707	2021	6.04	2022	2026	▂▂▂▂▂▂▂▂▂▂▂▂▂▂▃▃▃▃▃
61, CELL, V184, P4016, DOI 10.1016/j.cell.2021.05.021	2021	6.04	2022	2026	▂▂▂▂▂▂▂▂▂▂▂▂▂▂▃▃▃▃▃

### Keyword dynamics in DNGR-1 research

**Figure 10:**
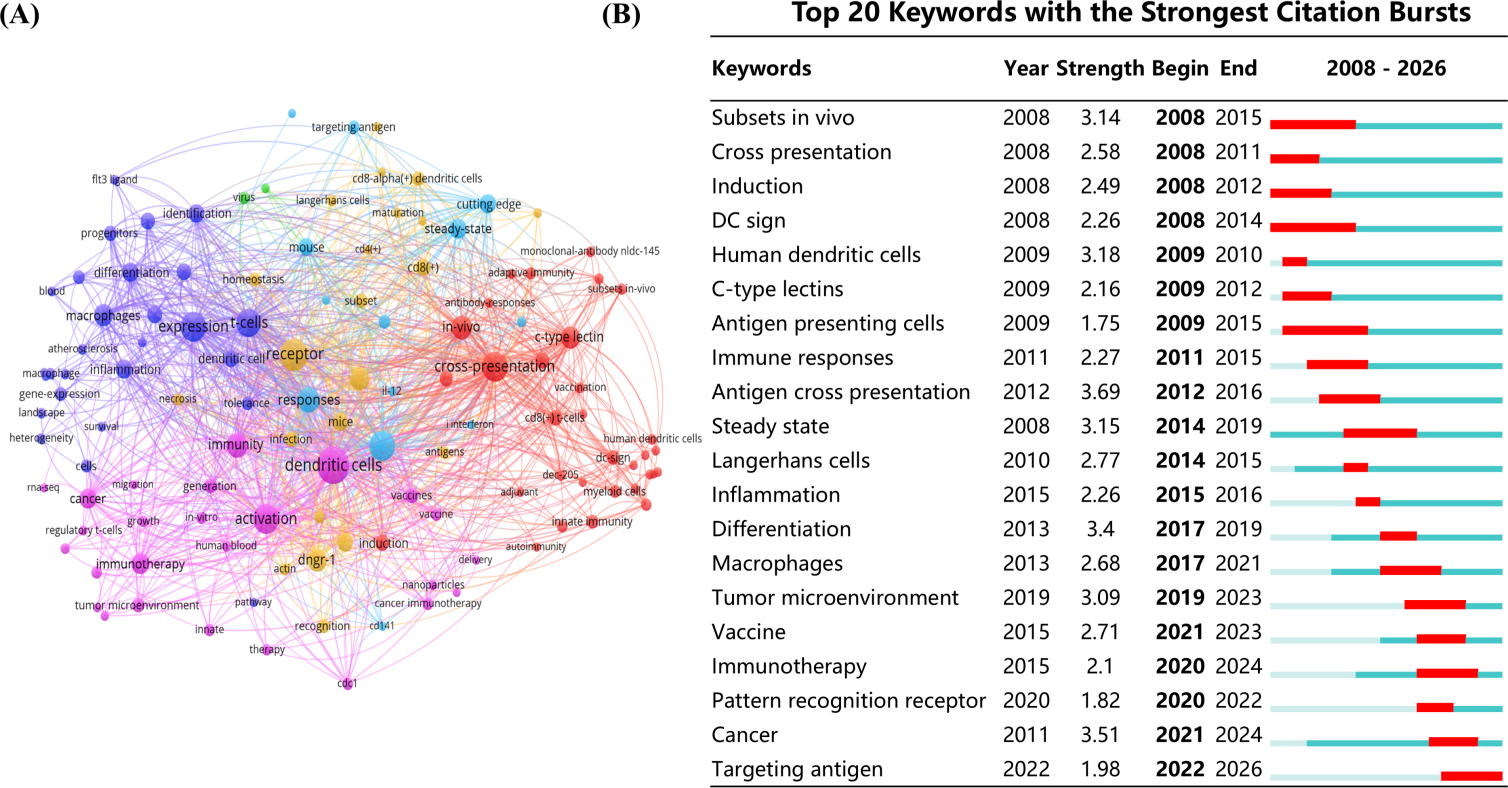
The visualization analysis of keywords. **(A)** Keyword network map. **(B)** Keywords with high citation explosiveness in articles related to DNGR-1 research.

The keyword co-occurrence network constructed using VOSviewer revealed DNGR-1 dynamic research trajectories from 2008 to 2026 (**Figure 10A**). A complementary burst detection analysis performed using CiteSpace (**Figure 10B**) identified 20 keywords that exhibited significant temporal occurrence patterns. In the temporal heatmap visualization, light hues denote latent keywords yet to emerge, while red segments highlight periods of high-frequency citation bursts (intensity > 2.5) and dark green areas indicate sustained scholarly attention. Notably, seven emerging high-impact keywords exceeded the 3.0 threshold for intensity: Subsets *in vivo* (intensity = 3.14), Human DC (intensity = 3.18), Antigen cross presentation (intensity = 3.69), Steady state (intensity = 3.15), Differentiation (intensity = 3.4), Cancer (intensity = 3.51)and Tumor microenvironment (intensity = 3.09), while the research frontier beyond 2019 is dominated by the “Tumor microenvironment” (2019-2023, intensity = 3.09) and “Cancer” (2021-2024, intensity = 3.51), together marking a paradigm shift in the application of molecular immunology in tumor therapy, particularly through lectin-mediated immunomodulatory strategies.

### Clinical progress analysis

Analysis of clinical studies reveals that CLEC9A serves as an important biomarker and therapeutic target in cancer immunotherapy and immune-mediated diseases. We categorize clinical evidence into observational associations (correlative findings) and functionally validated mechanisms (experimental interrogation of CLEC9A activity), based on eight studies retrieved from PubMed (**Table 6**). (1) Observational associations: CLEC9A as a prognostic biomarker: CLEC9A^+^ DC exhibit potent cross-presentation capabilities, with their enrichment in tumor microenvironments predicting enhanced responsiveness to immunotherapy in gastric and cervical cancers (63, 64). (2) Functionally demonstrated mechanisms: CLEC9A-mediated immune regulation. In melanoma and head and neck cancer patients, therapeutic interventions recruiting CLEC9A^+^BDCA3/CD141^+^ DC subsets correlated with improved anti-tumor immunity (65, 66). Conversely, anti-TNF therapy reduced synovial CLEC9A expression in psoriatic arthritis patients, suggesting a mechanism for reducing CD8-mediated inflammation (67). (3) translational applications: ex vivo CLEC9A^+^ DC generation. CLEC9A^+^ DC can be generated ex vivo from hematopoietic progenitors for DC-based immunotherapy (69), while elevated CLEC9A expression was associated with treatment failure in hemophilia A patients undergoing immune tolerance induction (68). The clinical investigation showed that CLEC9A could be a dual role as a predictive biomarker and immunoregulatory target.

**Table 6:** Clinical trials on DNGR-1.

Research area	Publication year	Title (Reference)
Observational associations: prognostic value	2014	Analysis of the transcriptome and immune function of monocytes during IFNα-based therapy in chronic HCV revealed induction of TLR7 responsiveness (62).
	2022	A multi-omics-based investigation of the prognostic and immunological impact of necroptosis-related mRNA in patients with cervical squamous carcinoma and adenocarcinoma (63).
	2025	Intestinal Subtype as a Biomarker of Response to Neoadjuvant Immunochemotherapy in Locally Advanced Gastric Adenocarcinoma: Insights from a Prospective Phase II Trial (64).
Mechanism: cross-presentation and CTable activation	2015	Arming the Melanoma Sentinel Lymph Node through Local Administration of CpG-B and GM-CSF: Recruitment and Activation of BDCA3/CD141(+) Dendritic Cells and Enhanced Cross-Presentation (65).
	2024	Dendritic cell effector mechanisms and tumor immune microenvironment infiltration define TLR8 modulation and PD-1 blockade (66).
Mechanism: therapeutic modulation	2016	Reduced CLEC9A expression in synovial tissue of psoriatic arthritis patients after adalimumab therapy (67).
	2025	Outcomes of immune tolerance induction with rituximab to eradicate high-titer inhibitor of hemophilia A: depicted by exponential decay model and the gene expression profile of different outcomes by RNA-sequencing (68).
Translational application	2014	The aryl hydrocarbon receptor antagonist StemRegenin 1 promotes human plasmacytoid and myeloid dendritic cell development from CD34^+^ hematopoietic progenitor cells (69).

## Discussion

### General information

In this study, we conducted a systematic literature search of the Web of Science database for articles on DNGR-1 published over the last 18 years (2008-2026). After excluding studies that did not meet the screening criteria, the scientometric study contained 326 articles published in 156 journals from 596 institutions in 42 countries/regions, with 12,657 co-citations.

DNGR-1 can provide a mechanism for cDC1 via binding to F-actin, which captures dead cell-associated antigens for cross-presentation to CD8^+^ T cells to promote tumor eradication (70). The selective expression of DNGR-1 by human and mouse cDC1 makes this receptor an attractive target for DC vaccines. The Clec9a antibody has been coupled to a range of different antigens derived from tumors and infectious agents, and the development of different Clec9a targeting modalities has become a hot research topic in recent years (71).

UK and USA ranked among the top ten most prolific countries, with a total of 135 publications from both countries, and their annual publication volume was broadly in line with the total annual publication volume. In particular, these two have contributed to more research in recent years, with nearly half of the articles published by them. China is the only developing country among the top ten most productive countries/regions, accounting for 16.56% of all publications. Australia accounts for one of the top institutions (5/10). We observed close co-operation between countries/regions and institutions. However, we found that co-operation between institutions was stronger than that between countries, implying that international co-operation should be strengthened.

The top 10 active authors published at least ten articles. Half of the top 10 active authors were from Australia, and the other half were from the UK. This finding implies that researchers from both countries played an important role and made significant contributions to DNGR-1 research. When considering co-cited authors, the top 10 authors with at least 67 co-citations made significant contributions to the field of DNGR-1 research. Sancho (228 co-citations) ranked first, followed by Caminschi, Irina (159 co-citations), and Poulin LF. (98 co-citations), respectively. Sancho et al. identified Clec9a as an endocytosis receptor that mediates internalization of bound antibodies and can target CD8α^+^ DC for antigen delivery *in vivo*. This allows cross-presentation to CD8^+^ T cells and, together with adjuvants, induces an effective CTL response that could cure mice with transplantable tumors, thus providing a novel strategy for cancer immunotherapy (12). They also found that Clec9a is essential for the cross-presentation of dying cowpox virus-infected (VACV-infected) cells to CD8^+^ T cells *in vitro*. Clec9a contributes to the generation of anti-VACV immunity after initial infection and vaccination. The ability of DNGR-1 to regulate the cross-presentation of viral antigens suggests that this form of anti-viral immunomodulation could be used for vaccination (24). Caminschi and Irina also concluded that Clec9a is a new DC subtype biomarker that crosses the species barrier and is a promising target for immunomodulation and for enhancing vaccine effectiveness (11). Targeting antigens to Clec9a in the absence of adjuvants results in effective humoral immunity. Many successful vaccines against pathogens or their toxins utilize humoral immunity as the primary effector mechanism. These approaches often use adjuvants or pathogenic materials to enhance the humoral response. However, the use of adjuvants has been associated with safety concerns. Therefore, inducing humoral immunity against Clec9a target antigens in the absence of adjuvants is an effective targeting strategy (72).

Frontiers in Immunology and the Journal of Immunology have more publications than other journals, suggesting that these journals are particularly interested in articles on DNGR-1. These data will help future scientists choose journals when submitting manuscripts related to DNGR-1.

Furthermore, after analyzing the top ten rankings of authors, co-citing authors, and co-cited references together, we found that Caminschi, Irina, Sancho, D., Lahoud, and Mireille H. were present in all three areas, suggesting that these three individuals and their teams are all Clec9a research areas of significant contributors and reliable collaborators.

### Knowledge base

Ten papers related to DNGR-1 were selected, specifically describing the mechanism of action of DNGR-1 between antigen cross-presentation and immunotherapy targeting, and its future development (shown **Table 4**).

DNGR-1 is a group V-type C-lectin-like receptor located on human chromosome 12 and mouse chromosome 6 in the NK complex (10, 12, 24). Human CLEC9A is highly expressed in the brain, thymus, and spleen tissues, but its expression in peripheral blood is highly restricted and detected only in BDCA3^+^ DC and a small number of CD14^+^CD16^-^ monocytes (**Table 4**, Rank-4) (10, 12, 73, 74). Human CLEC9A^+^BDCA3^+^ DC were phenotypically and functionally similar to the mouse CD8α^+^ DC. These cells can be produced in humanized mice and generated *in vitro*, providing novel methods for studying their properties and for immunotherapy (**Table 4**, Rank-9) (52). DC promote CD8^+^ T-cell immunity to viruses, bacteria, and tumor cells through cross-presentation (75). The absence of Batf3 affects the cross-presentation of antigens, indicating that cDC1 is closely related to the cross-presentation of antigens, which provides an important basis for further research on the relationship between the DC surface receptor Clec9a and cross-presentation (**Table 4**, Rank-6) (51). By studying the relationship between Clec9a and related ligands, which are normal cell components exposed when the cell membrane ruptures, and by using cryo-electron microscopy to observe the structure of the Clec9a complex with actin filaments (76), it was found that Clec9a binds to F-actin on the surface of dead cells (**Table 4**, Rank-5) (16). By staining the cells, it was found that even late stages of cell death, such as secondary necrosis, did not lead to the complete loss of F-actin in the cell cadaver, indicating that F-actin is the true agonist ligand of Clec9a, and the ability of necrotic cells to trigger Clec9a signaling via SYK is related to their F-actin content (**Table 4**, Rank-7) (14). In addition, the interaction of myosin II enhances the activation of Clec9a by F-actin (18). The Clec9a regulates the endocytic processing of antigens from necrotic cells, thereby promoting cross-presentation to cytotoxic T lymphocytes during viral infection in mice (**Table 4**, Rank-8) (17). In cross-presentation, DNGR-1 initiates a signaling pathway by recognizing exogenous cell-associated antigens. Upon phagocytosis, this pathway triggers NOX2-mediated pH modulation, reactive oxygen species (ROS) production, and aquaporin-3 activity, collectively facilitating perforin-2 maturation and phagosome-to-cytosol antigen transport. Perforin-2 then induces localized phagosome membrane rupture via lipid peroxidation (with ESCRT-III potentially repairing ruptured membranes). Finally, cytosolic antigens enter the classical MHC-I pathway-undergoing ubiquitination, proteasomal degradation, and TAP-dependent transport into the endoplasmic reticulum-for cross-presentation (**Table 4**, Rank-3) (13, 73, 77, 78). Furthermore, autophagy is an intracellular degradation system that transports cytoplasmic components to the lysosome (79), resulting in impaired integrity of the phagosomal membrane and the release of antigens into the cytosolic MHC-I pathway, thereby promoting cross-presentation (80).

Owing to its association with antigen cross-presentation, Clec9a can be targeted by antigens for tumor therapy. Mice with B16-melanoma were selected to study the therapeutic effects of DNGR-1-related antibodies. Exogenous antigen-targeting Clec9a, in combination with appropriate adjuvants, was also found to effectively cross-trigger CD8^+^ T cells, which can be used for preventive or therapeutic vaccination against mouse tumors (**Table 4**, Rank-2) (12). Induction of type I IFN in tumors can overcome innate immune resistance and activate anti-tumor adaptive immunity, promoting cDC1 cross-priming and CD8^+^ T cell reactivation (81). Clec9a can be used as a target for vaccine enhancement (**Table 4**, Rank-1) and can promote the cross-presentation and proliferation of Ag-specific transgenic CD8^+^ and CD4^+^ T cells, even in the absence of adjuvants, and promote the efficient development of follicular helper T cells (**Table 4**, Rank-10) (11, 39). This tumor antigen vaccine targeting Clec9a, which targets a critical subset of DC required to initiate tumor-specific immune responses, thereby reducing off-target effects while maximizing potential efficacy, will be widely applicable to many cancer types and suitable for use in combination with a variety of other therapies to enhance tumor immunogenicity (82, 83).

### Research hotspots

To further discuss the research hotspots on DNGR-1, we used CiteSpace to examine co-cited references. As shown in **Figure 9**, early research has focused on #2 (BDCA3), #3 (Myeloid Cells), #7 (DC NK Lectin Group Receptor-1), and #8 (C-type Lectins). Recent studies have focused on #0 (Adaptive Immunity), #5 (Spatial Transcrptomics), and #11 (System Biology).

Early research primarily focused on fundamental studies of DNGR-1 molecular characteristics, expression, and immune targets. In mice, Clec9a exhibits high expression in CD8^+^ cDC1 and low expression in pDC. Human CLEC9A is exclusively expressed on BDCA3^+^ DC and a minor subset of CD14^+^CD16^-^ monocytes (10, 12). Functional validation in mouse models revealed that targeting Clec9a mediates cross-presentation of tumor antigens, eliciting potent antitumor responses and serving as a target to enhance vaccine efficacy by simultaneously boosting both antibody and T cell responses against antigens (11, 12). Subsequent human cell experiments further confirmed CLEC9A role in mediating antigen presentation by BDCA3^+^ DC, establishing it as an ideal immunological target for enhancing the immunogenicity of malignant tumor vaccines (41).

Further investigation revealed its signaling pathway: DNGR-1 initiates signal transduction to activate SYK and NADPH oxidase, inducing phagosome lysis and thereby promoting cross-presentation of necrosis-associated antigens to regulate adaptive immunity (60). CLEC9A^+^ DC function as antigen-presenting cells, enabling *in situ* T-cell activation in multiple myeloma. Enhancing the function of CLEC9A^+^ DC within the tumor microenvironment can improve the durability of immunotherapy for multiple myeloma (84). Furthermore, extracellular protein secretory glycocalyx protein (sGSN) reduces DNGR-1 binding to F-actin, thereby inhibiting cDC1 cross-presentation of death cell-associated antigens, providing a novel regulatory target for anti-tumor immunotherapy (61). Single-cell transcriptomics identified a novel immune cell subtype, CLEC9A^+^ DC, in nasopharyngeal carcinoma. This discovery confirmed a significant association between CLEC9A^+^ DC expression and favorable patient survival, suggesting its potential role in inducing antitumor immune responses (85). Spatial transcriptomics research holds promise for elucidating the spatial heterogeneity of the tumor microenvironment (84). Current research focuses on regulating DNGR-1-associated adaptive immunity. Single-cell transcriptomics reveals specific interactions between tumors and their microenvironments, identifying novel immune cell subtypes and targets. However, clinical translation requires further validation.

### Clinical research on CLEC9A in human disease

In recent years, CLEC9A has emerged as a critical biomarker and therapeutic target across multiple clinical data, particularly in cancer immunotherapy and immune-mediated diseases. A total of eight clinical data were retrieved from the PubMed database (**Table 6**), which can be categorized into the following areas based on their research themes. Analysis of these studies highlights key findings: (1) The prognostic value of CLEC9A in cancer immunotherapy (observational associations). Clinical evidence demonstrates that CLEC9A expression on DC serves as a significant predictor of treatment response and patient prognosis. In cervical squamous carcinoma and adenocarcinoma (CESC), CLEC9A was identified as one of three necroptosis-related mRNAs constituting an independent prognostic risk score model, with the low-risk subgroup (characterized by distinct CLEC9A expression patterns) showing enhanced immune cell infiltration and better response to immune checkpoint inhibitors (63). Similarly, in gastric adenocarcinoma, enrichment of CLEC9A^+^ DC in the tumor microenvironment was associated with enhanced responsiveness to neoadjuvant immunochemotherapy, and a machine learning algorithm integrating CLEC9A^+^ DC transcriptomic features accurately predicted treatment efficacy across multiple independent cohorts (64). (2) CLEC9A^+^ DC as mediators of anti-tumor immunity (mechanism: cross-presentation and CTL activation). The recruitment and activation of CLEC9A-expressing BDCA3/CD141^+^ DC subsets have been demonstrated to enhance cross-presentation and CTL responses in cancer patients. In melanoma patients receiving combined CpG-B and GM-CSF therapy, increased frequencies of sentinel lymph node-resident BDCA3/CD141^+^ DC subsets expressing CLEC9A correlated with enhanced ex vivo cross-presenting capacity (65). Furthermore, in head and neck squamous cell carcinoma patients treated with TLR8 agonism combined with anti-PD-1 blockade, dual therapy resulted in a specific increase in CLEC9A^+^XCR1^+^ DC, which are specialized in cross-presentation, alongside upregulation of immune effector genes including MYD88 and SYK (66). (3) Modulation of CLEC9A expression by therapeutic interventions (mechanism: therapeutic modulation). Anti-TNF therapy has been shown to specifically reduce CLEC9A expression in inflammatory conditions. In psoriatic arthritis patients, adalimumab treatment significantly decreased synovial tissue CLEC9A protein expression compared with placebo, suggesting that downregulation of synovial CLEC9A may represent a novel mechanism by which anti-TNF therapy reduces CD8^+^ T-mediated inflammation (67). Conversely, in hemophilia A patients undergoing immune tolerance induction with rituximab, CLEC9A was identified as an upregulated differentially expressed gene in treatment failure subjects, implicating humoral immune response pathways in poor therapeutic outcomes (68). (4) Ex vivo generation of CLEC9A^+^ DC for immunotherapy (translational applications). The aryl hydrocarbon receptor antagonist StemRegenin 1 has been successfully employed to generate clinical-scale numbers of functional BDCA3^+^CLEC9A^+^ myeloid DC from CD34^+^ hematopoietic progenitor cells. These ex vivo-generated DC demonstrated phenotypic and functional properties comparable to peripheral blood DC, including potent allogeneic T-cell stimulatory capacity and antigen-specific T-cell activation, providing an alternative to monocyte-derived DC for cancer immunotherapy applications (69). Accumulating clinical evidence positions CLEC9A as a critical regulator of DC-mediated immune responses with significant translational potential as both a predictive biomarker and a therapeutic target in cancer immunotherapy and immune-mediated diseases.

### Strengths and limitations

This study integrates WOSCC and Scopus data, employing CiteSpace, VOSviewer, and Bibliometrix software to explore DNGR-1 research hotspots from multiple dimensions. Subsequently, the PubMed database was utilized to analyze clinical research progress, exploring future development directions and providing comprehensive guidance for clinicians and scholars in this field. The aim is to promote disciplinary advancement and knowledge accumulation, driving both the depth and breadth of DNGR-1 research. Notably, this systematic literature search on DNGR-1 gives tremendous information on the expanding research towards immunology, immunotherapy, and cancer therapy. And to a researcher, the bibliography mapping gives a great view of future collaboration and ideas. This study presents the following limitations: Firstly, potential omissions exist in the included literature. The exclusive retrieval of English-language publications introduces language bias, meaning the identified literature may not comprehensively cover all DNGR-1 research. Secondly, due to the cutoff date for literature retrieval (January 2026), subsequent studies could not be included in the analysis, which may limit the comprehensive understanding of research trends throughout 2026. Thirdly, this article is not for general readers since it’s so in-depth, and an additional limitation includes this is a systematic literature search. With the continuous advancement of research methodologies and the iterative upgrading of analytical tools, it is anticipated that more advanced and systematic research paradigms will emerge, further uncovering the underlying mechanisms and application potential of DNGR-1 in immune regulation and disease intervention.

## Conclusion

In conclusion, research on DNGR-1 has become increasingly in-depth. Current research hotspots in this field mainly focus on anti-tumor immunotherapy and DC-targeted vaccination. In-depth exploration and elucidation of the specific mechanism of action of DNGR-1, studies of its relevance in infection and cancer, and the development of effective clinical applications are the focus of future research.

## Data Availability

The original contributions presented in the study are included in the article/**Supplementary Material**. Further inquiries can be directed to the corresponding author.

## References

[1] Böttcher JP Reis e Sousa C . The role of type 1 conventional dendritic cells in cancer immunity. Trends Cancer. (2018) 4:784–92. doi: 10.1016/j.trecan.2018.09.001, PMID: 30352680 PMC6207145

[2] Kvedaraite E Ginhoux F . Human dendritic cells in cancer. Sci Immunol. (2022) 7:eabm9409. doi: 10.1126/sciimmunol.abm9409. PMID: 35363544

[3] Guilliams M Ginhoux F Jakubzick C Naik SH Onai N Schraml BU . Dendritic cells, monocytes and macrophages: a unified nomenclature based on ontogeny. Nat Rev Immunol. (2014) 14:571–8. doi: 10.1038/nri3712. PMID: 25033907 PMC4638219

[4] Greene TT Jo YR Zuniga EI . Infection and cancer suppress pDC derived IFN-I. Curr Opin Immunol. (2020) 66:114–22. doi: 10.1016/j.coi.2020.08.001. PMID: 32947131 PMC8526282

[5] Ohara RA Murphy KM . Recent progress in type 1 classical dendritic cell cross-presentation - cytosolic, vacuolar, or both? Curr Opin Immunol. (2023) 83:102350. doi: 10.1016/j.coi.2023.102350. PMID: 37276818 PMC12013855

[6] Villar J Segura E . The more, the merrier: DC3s join the human dendritic cell family. Immunity. (2020) 53:233–5. doi: 10.1016/j.immuni.2020.07.014. PMID: 32814019

[7] Liu Z Wang H Li Z Dress RJ Zhu Y Zhang S . Dendritic cell type 3 arises from Ly6C(+) monocyte-dendritic cell progenitors. Immunity. (2023) 56:1761–1777.e6. doi: 10.1016/j.immuni.2023.07.001. PMID: 37506694

[8] Rawat K Tewari A Li X Mara AB King WT Gibbings SL . CCL5-producing migratory dendritic cells guide CCR5+ monocytes into the draining lymph nodes. J Exp Med. (2023) 220:e20222129. doi: 10.1084/jem.20222129. PMID: 36946983 PMC10072223

[9] Romero P Banchereau J Bhardwaj N Cockett M Disis ML Dranoff G . The Human Vaccines Project: A roadmap for cancer vaccine development. Sci Transl Med. (2016) 8:334ps9. doi: 10.1126/scitranslmed.aaf0685. PMID: 27075624

[10] Huysamen C Willment JA Dennehy KM Brown GD . CLEC9A is a novel activation C-type lectin-like receptor expressed on BDCA3+ dendritic cells and a subset of monocytes. J Biol Chem. (2008) 283:16693–701. doi: 10.1074/jbc.m709923200. PMID: 18408006 PMC2562446

[11] Caminschi I Proietto AI Ahmet F Kitsoulis S Shin Teh J Lo JC . The dendritic cell subtype-restricted C-type lectin Clec9A is a target for vaccine enhancement. Blood. (2008) 112:3264–73. doi: 10.1182/blood-2008-05-155176. PMID: 18669894 PMC2569177

[12] Sancho D Mourão-Sá D Joffre OP Schulz O Rogers NC Pennington DJ . Tumor therapy in mice via antigen targeting to a novel, DC-restricted C-type lectin. J Clin Invest. (2008) 118:2098–110. doi: 10.1172/jci34584. PMID: 18497879 PMC2391066

[13] Sancho D Joffre OP Keller AM Rogers NC Martínez D Hernanz-Falcón P . Identification of a dendritic cell receptor that couples sensing of necrosis to immunity. Nature. (2009) 458:899–903. doi: 10.1038/nature07750. PMID: 19219027 PMC2671489

[14] Ahrens S Zelenay S Sancho D Hanč P Kjær S Feest C . F-actin is an evolutionarily conserved damage-associated molecular pattern recognized by DNGR-1, a receptor for dead cells. Immunity. (2012) 36:635–45. doi: 10.1016/j.immuni.2012.03.008. PMID: 22483800

[15] Poulin LF Reyal Y Uronen-Hansson H Schraml BU Sancho D Murphy KM . DNGR-1 is a specific and universal marker of mouse and human Batf3-dependent dendritic cells in lymphoid and nonlymphoid tissues. Blood. (2012) 119:6052–62. doi: 10.1182/blood-2012-01-406967. PMID: 22442345

[16] Zhang JG Czabotar PE Policheni AN Caminschi I Wan SS Kitsoulis S . The dendritic cell receptor Clec9A binds damaged cells via exposed actin filaments. Immunity. (2012) 36:646–57. doi: 10.1016/j.immuni.2012.03.009. PMID: 22483802

[17] Zelenay S Keller AM Whitney PG Schraml BU Deddouche S Rogers NC . The dendritic cell receptor DNGR-1 controls endocytic handling of necrotic cell antigens to favor cross-priming of CTLs in virus-infected mice. J Clin Invest. (2012) 122:1615–27. doi: 10.1172/jci60644. PMID: 22505458 PMC3336984

[18] Schulz O Hanč P Böttcher JP Hoogeboom R Diebold SS Tolar P . Myosin II synergizes with F-Actin to promote DNGR-1-dependent cross-presentation of dead cell-associated antigens. Cell Rep. (2018) 24:419–28. doi: 10.1016/j.celrep.2018.06.038. PMID: 29996102 PMC6057488

[19] Macri C Dumont C Panozza S Lahoud MH Caminschi I Villadangos JA . Antibody-mediated targeting of antigen to C-type lectin-like receptors Clec9A and Clec12A elicits different vaccination outcomes. Mol Immunol. (2017) 81:143–50. doi: 10.1016/j.molimm.2016.12.010. PMID: 27978488

[20] Tullett KM Leal Rojas IM Minoda Y Tan PS Zhang JG Smith C . Targeting CLEC9A delivers antigen to human CD141(+) DC for CD4(+) and CD8(+)T cell recognition. JCI Insight. (2016) 1:e87102. doi: 10.1172/jci.insight.87102. PMID: 27699265 PMC5033826

[21] Yan Z Wu Y Du J Li G Wang S Cao W . A novel peptide targeting Clec9a on dendritic cell for cancer immunotherapy. Oncotarget. (2016) 7:40437–50. doi: 10.18632/oncotarget.9624. PMID: 27250027 PMC5130018

[22] Ghinnagow R de Meester J Cruz LJ Aspord C Corgnac S Macho-Fernandez E . Co-delivery of the NKT agonist α-galactosylceramide and tumor antigens to cross-priming dendritic cells breaks tolerance to self-antigens and promotes antitumor responses. Oncoimmunology. (2017) 6:e1339855. doi: 10.1080/2162402x.2017.1339855. PMID: 28932640 PMC5599097

[23] Park HY Tan PS Kavishna R Ker A Lu J Chan CEZ . Enhancing vaccine antibody responses by targeting Clec9A on dendritic cells. NPJ Vaccines. (2017) 2:31. doi: 10.1038/s41541-017-0033-5. PMID: 29263886 PMC5674066

[24] Iborra S Izquierdo HM Martínez-López M Blanco-Menéndez N Reis e Sousa C Sancho D . The DC receptor DNGR-1 mediates cross-priming of CTLs during vaccinia virus infection in mice. J Clin Invest. (2012) 122:1628–43. doi: 10.1172/jci60660. PMID: 22505455 PMC3336985

[25] Del Fresno C Saz-Leal P Enamorado M Wculek SK Martínez-Cano S Blanco-Menéndez N . DNGR-1 in dendritic cells limits tissue damage by dampening neutrophil recruitment. Science. (2018) 362:351–6. doi: 10.1126/science.aan8423. PMID: 30337411

[26] Shang R Pan T Wang F Jin H Nan X Song C . Current and future trends of acupuncture as an adjuvant therapy in cancer: A bibliometric and visual analysis. Medicine. (2024) 103:p e38663. doi: 10.1097/md.0000000000038663. PMID: 39029068 PMC11398740

[27] Pei Z Chen S Ding L Liu J Cui X Li F . Current perspectives and trend of nanomedicine in cancer: a review and bibliometric analysis. J Control Release. (2022) 352:211–41. doi: 10.1016/j.jconrel.2022.10.023. PMID: 36270513

[28] Wei N Xu Y Li Y Shi J Zhang X You Y . A bibliometric analysis of T cell and atherosclerosis. Front Immunol. (2022) 13:948314. doi: 10.3389/fimmu.2022.948314. PMID: 36311729 PMC9606647

[29] Chen C Hu Z Liu S Tseng H . Emerging trends in regenerative medicine: a scientometric analysis in CiteSpace. Expert Opin Biol Ther. (2012) 12:593–608. doi: 10.1517/14712598.2012.674507. PMID: 22443895

[30] Jongbloed SL Kassianos AJ McDonald KJ Clark GJ Ju X Angel CE . Human CD141+ (BDCA-3)+ dendritic cells (DCs) represent a unique myeloid DC subset that cross-presents necrotic cell antigens. J Exp Med. (2010) 207:1247–60. doi: 10.1084/jem.20092140. PMID: 20479116 PMC2882828

[31] Joffre OP Sancho D Zelenay S Keller AM Reis e Sousa C . Efficient and versatile manipulation of the peripheral CD4+ T-cell compartment by antigen targeting to DNGR-1/CLEC9A. Eur J Immunol. (2010) 40:1255–65. doi: 10.1002/eji.201040419. PMID: 20333625 PMC3064981

[32] Hanč P Fujii T Iborra S Yamada Y Huotari J Schulz O . Structure of the complex of F-actin and DNGR-1, a C-type lectin receptor involved in dendritic cell cross-presentation of dead cell-associated antigens. Immunity. (2015) 42:839–49. doi: 10.1016/j.immuni.2015.04.009, PMID: 25979418 PMC5066845

[33] Schraml BU van Blijswijk J Zelenay S Whitney PG Filby A Acton SE . Genetic tracing via DNGR-1 expression history defines dendritic cells as a hematopoietic lineage. Cell. (2013) 154:843–58. doi: 10.1016/j.cell.2013.07.014. PMID: 23953115

[34] Caminschi I Shortman K . Boosting antibody responses by targeting antigens to dendritic cells. Trends Immunol. (2012) 33:71–7. doi: 10.1016/j.it.2011.10.007. PMID: 22153931

[35] Caminschi I Vremec D Ahmet F Lahoud MH Villadangos JA Murphy KM . Antibody responses initiated by Clec9A-bearing dendritic cells in normal and Batf3(-/-) mice. Mol Immunol. (2012) 50:9–17. doi: 10.1016/j.molimm.2011.11.008. PMID: 22209163

[36] Durant LR Pereira C Boakye A Makris S Kausar F Goritzka M . DNGR-1 is dispensable for CD8+ T-cell priming during respiratory syncytial virus infection. Eur J Immunol. (2014) 44:2340–8. doi: 10.1002/eji.201444454. PMID: 24777856

[37] Gou S Liu W Wang S Chen G Chen Z Qiu L . Engineered nanovaccine targeting Clec9a(+) dendritic cells remarkably enhances the cancer immunotherapy effects of STING agonist. Nano Lett. (2021) 21:9939–50. doi: 10.1021/acs.nanolett.1c03243. PMID: 34779631

[38] Idoyaga J Lubkin A Fiorese C Lahoud MH Caminschi I Huang Y . Comparable T helper 1 (Th1) and CD8 T-cell immunity by targeting HIV gag p24 to CD8 dendritic cells within antibodies to Langerin, DEC205, and Clec9A. Proc Natl Acad Sci USA. (2011) 108:2384–9. doi: 10.1073/pnas.1019547108. PMID: 21262813 PMC3038758

[39] Lahoud MH Ahmet F Kitsoulis S Wan SS Vremec D Lee CN . Targeting antigen to mouse dendritic cells via Clec9A induces potent CD4 T cell responses biased toward a follicular helper phenotype. J Immunol. (2011) 187:842–50. doi: 10.4049/jimmunol.1101176. PMID: 21677141

[40] Picco G Beatson R Taylor-Papadimitriou J Burchell JM . Targeting DNGR-1 (CLEC9A) with antibody/MUC1 peptide conjugates as a vaccine for carcinomas. Eur J Immunol. (2014) 44:1947–55. doi: 10.1002/eji.201344076. PMID: 24648154 PMC4209794

[41] Schreibelt G Klinkenberg LJ Cruz LJ Tacken PJ Tel J Kreutz M . The C-type lectin receptor CLEC9A mediates antigen uptake and (cross-)presentation by human blood BDCA3+ myeloid dendritic cells. Blood. (2012) 119:2284–92. doi: 10.1182/blood-2011-08-373944. PMID: 22234694

[42] Schuette V Burgdorf S . The ins-and-outs of endosomal antigens for cross-presentation. Curr Opin Immunol. (2014) 26:63–8. doi: 10.1016/j.coi.2013.11.001. PMID: 24556402

[43] Tullett KM Lahoud MH Radford KJ . Harnessing human cross-presenting CLEC9A(+)XCR1(+) dendritic cells for immunotherapy. Front Immunol. (2014) 5:239. doi: 10.3389/fimmu.2014.00239. PMID: 24904587 PMC4033245

[44] Gou S Wang S Liu W Chen G Zhang D Du J . Adjuvant-free peptide vaccine targeting Clec9a on dendritic cells can induce robust antitumor immune response through Syk/IL-21 axis. Theranostics. (2021) 11:7308–21. doi: 10.7150/thno.56406. PMID: 34158852 PMC8210616

[45] Zeng B Middelberg AP Gemiarto A Macdonald K Baxter AG Talekar M . Self-adjuvanting nanoemulsion targeting dendritic cell receptor Clec9A enables antigen-specific immunotherapy. J Clin Invest. (2018) 128:1971–84. doi: 10.1172/jci96791. PMID: 29485973 PMC5919883

[46] Lam PY Kobayashi T Soon M Zeng B Dolcetti R Leggatt G . NKT cell-driven enhancement of antitumor immunity induced by Clec9a-targeted tailorable nanoemulsion. Cancer Immunol Res. (2019) 7:952–62. doi: 10.1158/2326-6066.cir-18-0650. PMID: 31053598

[47] Vu MN Pilkington EH Lee WS Tan HX Davis TP Truong NP . Engineered ferritin nanoparticle vaccines enable rapid screening of antibody functionalization to boost immune responses. Adv Healthc Mater. (2023) 12:e2202595. doi: 10.1002/adhm.202202595. PMID: 36786027 PMC11469303

[48] van Lint S van Parys A van den Eeckhout B Vandamme N Plaisance S Verhee A . A bispecific Clec9A-PD-L1 targeted type I interferon profoundly reshapes the tumor microenvironment towards an antitumor state. Mol Cancer. (2023) 22:191. doi: 10.1186/s12943-023-01908-6. PMID: 38031106 PMC10685570

[49] Cheang NYZ Tan KS Tan PS Purushotorma K Yap WC Tullett KM . Single-shot dendritic cell targeting SARS-CoV-2 vaccine candidate induces broad, durable and protective systemic and mucosal immunity in mice. Mol Ther. (2024) 32:2299–315. doi: 10.1016/j.ymthe.2024.05.003. PMID: 38715364 PMC11286822

[50] Holley CL Monteleone M Fisch D Libert AES Ju RJ Choi JH . Pyroptotic cell corpses are crowned with F-actin-rich filopodia that engage CLEC9A signaling in incoming dendritic cells. Nat Immunol. (2025) 26:42–52. doi: 10.1038/s41590-024-02024-3. PMID: 39633178 PMC11695261

[51] Hildner K Edelson BT Purtha WE Diamond M Matsushita H Kohyama M . Batf3 deficiency reveals a critical role for CD8alpha+ dendritic cells in cytotoxic T cell immunity. Science. (2008) 322:1097–100. doi: 10.1126/science.1164206. PMID: 19008445 PMC2756611

[52] Poulin LF Salio M Griessinger E Anjos-Afonso F Craciun L Chen JL . Characterization of human DNGR-1+ BDCA3+ leukocytes as putative equivalents of mouse CD8alpha+ dendritic cells. J Exp Med. (2010) 207:1261–71. doi: 10.1084/jem.20092618. PMID: 20479117 PMC2882845

[53] Bachem A Güttler S Hartung E Ebstein F Schaefer M Tannert A . Superior antigen cross-presentation and XCR1 expression define human CD11c+CD141+ cells as homologues of mouse CD8+ dendritic cells. J Exp Med. (2010) 207:1273–81. doi: 10.1084/jem.20100348, PMID: 20479115 PMC2882837

[54] Crozat K Guiton R Contreras V Feuillet V Dutertre CA Ventre E . The XC chemokine receptor 1 is a conserved selective marker of mammalian cells homologous to mouse CD8α+ dendritic cells. J Exp Med. (2010) 207:1283–92. doi: 10.1084/jem.20100223, PMID: 20479118 PMC2882835

[55] Haniffa M Shin A Bigley V McGovern N Teo P See P . Human tissues contain CD141^hi^ cross-presenting dendritic cells with functional homology to mouse CD103^+^ nonlymphoid dendritic cells. Immunity. (2012) 37:60–73. doi: 10.1016/j.immuni.2012.04.012, PMID: 22795876 PMC3476529

[56] Villani AC Satija R Reynolds G Sarkizova S Shekhar K Fletcher J . Single-cell RNA-seq reveals new types of human blood dendritic cells, monocytes, and progenitors. Science. (2017) 356:eaah4573. doi: 10.1126/science.aah4573, PMID: 28428369 PMC5775029

[57] Spranger S Dai D Horton B Gajewski TF . Tumor-Residing Batf3 Dendritic Cells Are Required for Effector T Cell Trafficking and Adoptive T Cell Therapy. Cancer Cell. (2017) 31:711–723.e4. doi: 10.1016/j.ccell.2017.04.003, PMID: 28486109 PMC5650691

[58] Cabeza-Cabrerizo M Cardoso A Minutti CM Pereira da Costa M Reis e Sousa C . Dendritic Cells Revisited. Annu Rev Immunol. (2021) 39:131–66. doi: 10.1146/annurev-immunol-061020-053707, PMID: 33481643

[59] Böttcher JP Bonavita E Chakravarty P Blees H Cabeza-Cabrerizo M Sammicheli S . NK cells stimulate recruitment of cDC1 into the tumor microenvironment promoting cancer immune control. Cell. (2018) 172:1022–1037.e14. doi: 10.1016/j.cell.2018.01.004, PMID: 29429633 PMC5847168

[60] Canton J Blees H Henry CM Buck MD Schulz O Rogers NC . The receptor DNGR-1 signals for phagosomal rupture to promote cross-presentation of dead-cell-associated antigens. Nat Immunol. (2021) 22:140–53. doi: 10.1038/s41590-020-00824-x. PMID: 33349708 PMC7116638

[61] Giampazolias E Schulz O Lim KHJ Rogers NC Chakravarty P Srinivasan N . Secreted gelsolin inhibits DNGR-1-dependent cross-presentation and cancer immunity. Cell. (2021) 184:4016–4031.e22. doi: 10.1016/j.cell.2021.05.021. PMID: 34081922 PMC8320529

[62] Hou J Groothuismink ZMA Koning L Roomer R van IJcken WFJ Kreefft K . Analysis of the transcriptome and immune function of monocytes during IFNα-based therapy in chronic HCV revealed induction of TLR7 responsiveness. Antiviral Res. (2014) 109:116–24. doi: 10.1016/j.antiviral.2014.06.020. PMID: 25014880

[63] Zou J Lin Z Jiao W Chen J Lin L Zhang F . A multi-omics-based investigation of the prognostic and immunological impact of necroptosis-related mRNA in patients with cervical squamous carcinoma and adenocarcinoma. Sci Rep. (2022) 12:16773. doi: 10.1038/s41598-022-20566-0. PMID: 36202899 PMC9537508

[64] Wang L Sun M Li J Wan L Tan Y Tian S . Intestinal subtype as a biomarker of response to neoadjuvant immunochemotherapy in locally advanced gastric adenocarcinoma: Insights from a prospective phase II trial. Clin Cancer Res. (2025) 31:74–86. doi: 10.1158/1078-0432.ccr-24-2436. PMID: 39495175

[65] Sluijter BJ van den Hout MF Koster BD van Leeuwen PA Schneiders FL van de Ven R . Arming the melanoma sentinel lymph node through local administration of CpG-B and GM-CSF: Recruitment and activation of BDCA3/CD141(+) dendritic cells and enhanced cross-presentation. Cancer Immunol Res. (2015) 3:495–505. doi: 10.1158/2326-6066.cir-14-0165. PMID: 25633713

[66] Ruiz-Torres DA Wise JF Zhao BY Oliveira-Costa JP Cavallaro S Sadow PM . Dendritic cell effector mechanisms and tumor immune microenvironment infiltration define TLR8 modulation and PD-1 blockade. Front Immunol. (2024) 15:1440530. doi: 10.3389/fimmu.2024.1440530. PMID: 39697344 PMC11652363

[67] Ramos MI Teunissen MB Helder B Aarrass S de Hair MJ van Kuijk AW . Reduced CLEC9A expression in synovial tissue of psoriatic arthritis patients after adalimumab therapy. Rheumatol (Oxford). (2016) 55:1575–84. doi: 10.1093/rheumatology/kew204. PMID: 27179104

[68] Li Z Tang Y Chen Z Liu G Yao W Li G . Outcomes of immune tolerance induction with rituximab to eradicate high-titer inhibitor of hemophilia A: depicted by exponential decay model and the gene expression profile of different outcomes by RNA-sequencing. J Thromb Haemost. (2025) 23:2436–48. doi: 10.1016/j.jtha.2025.04.015. PMID: 40286913

[69] Thordardottir S Hangalapura BN Hutten T Cossu M Spanholtz J Schaap N . The aryl hydrocarbon receptor antagonist StemRegenin 1 promotes human plasmacytoid and myeloid dendritic cell development from CD34+ hematopoietic progenitor cells. Stem Cells Dev. (2014) 23:955–67. doi: 10.1089/scd.2013.0521. PMID: 24325394

[70] Masterman KA Haigh OL Tullett KM Leal-Rojas IM Walpole C Pearson FE . Human CLEC9A antibodies deliver NY-ESO-1 antigen to CD141(+) dendritic cells to activate naïve and memory NY-ESO-1-specific CD8(+) T cells. J Immunother Cancer. (2020) 8:A368. doi: 10.1136/jitc-2020-sitc2020.0612. PMID: 32737142 PMC7394304

[71] Macri C Jenika D Ouslinis C Mintern JD . Targeting dendritic cells to advance cross-presentation and vaccination outcomes. Semin Immunol. (2023) 68:101762. doi: 10.1016/j.smim.2023.101762. PMID: 37167898

[72] Li J Ahmet F Sullivan LC Brooks AG Kent SJ de Rose R . Antibodies targeting Clec9A promote strong humoral immunity without adjuvant in mice and non-human primates. Eur J Immunol. (2015) 45:854–64. doi: 10.1002/eji.201445127. PMID: 25487143

[73] Rodríguez-Silvestre P Laub M Krawczyk PA Davies AK Schessner JP Parveen R . Perforin-2 is a pore-forming effector of endocytic escape in cross-presenting dendritic cells. Science. (2023) 380:1258–65. doi: 10.1126/science.adg8802. PMID: 37347855 PMC7614779

[74] van der Aa E van Montfoort N Woltman AM . BDCA3(+)CLEC9A(+) human dendritic cell function and development. Semin Cell Dev Biol. (2015) 41:39–48. doi: 10.1016/j.semcdb.2014.05.016. PMID: 24910448

[75] Blander JM . Different routes of MHC-I delivery to phagosomes and their consequences to CD8 T cell immunity. Semin Immunol. (2023) 66:101713. doi: 10.1016/j.smim.2023.101713. PMID: 36706521 PMC10023361

[76] Hanč P Schulz O Fischbach H Martin SR Kjær S Reis e Sousa C . A pH- and ionic strength-dependent conformational change in the neck region regulates DNGR-1 function in dendritic cells. EMBO J. (2016) 35:2484–97. doi: 10.15252/embj.201694695, PMID: 27753620 PMC5109244

[77] Dingjan I Verboogen DR Paardekooper LM Revelo NH Sittig SP Visser LJ . Lipid peroxidation causes endosomal antigen release for cross-presentation. Sci Rep. (2016) 6:22064. doi: 10.1038/srep22064. PMID: 26907999 PMC4764948

[78] Rawat K Jakubzick CV . Channeling antigens to CD8(+) T cells. Science. (2023) 380:1218–9. doi: 10.1126/science.adi5711. PMID: 37347866 PMC10589910

[79] van Kaer L Parekh VV Postoak JL Wu L . Role of autophagy in MHC class I-restricted antigen presentation. Mol Immunol. (2019) 113:2–5. doi: 10.1016/j.molimm.2017.10.021. PMID: 29126597 PMC5940586

[80] Policastro LL Ibañez IL Notcovich C Duran HA Podhajcer OL . The tumor microenvironment: characterization, redox considerations, and novel approaches for reactive oxygen species-targeted gene therapy. Antioxid Redox Signal. (2013) 19:854–95. doi: 10.1089/ars.2011.4367. PMID: 22794113

[81] Liang Y Hannan R Fu YX . Type I IFN activating type I dendritic cells for antitumor immunity. Clin Cancer Res. (2021) 27:3818–24. doi: 10.1158/1078-0432.ccr-20-2564. PMID: 33692027

[82] Zhou Y Slone N Chrisikos TT Kyrysyuk O Babcock RL Medik YB . Vaccine efficacy against primary and metastatic cancer with *in vitro*-generated CD103(+) conventional dendritic cells. J Immunother Cancer. (2020) 8:e000474. doi: 10.4049/jimmunol.204.supp.91.4. PMID: 32273347 PMC7254126

[83] Lahoud MH Radford KJ . Enhancing the immunogenicity of cancer vaccines by harnessing CLEC9A. Hum Vaccin Immunother. (2022) 18:1873056. doi: 10.1080/21645515.2021.1873056. PMID: 33625943 PMC8920153

[84] Hammerl D Martens JWM Timmermans M Smid M Trapman-Jansen AM Foekens R . Spatial immunophenotypes predict response to anti-PD1 treatment and capture distinct paths of T cell evasion in triple negative breast cancer. Nat Commun. (2021) 12:5668. doi: 10.1038/s41467-021-25962-0. PMID: 34580291 PMC8476574

[85] Chen YP Yin JH Li WF Li HJ Chen DP Zhang CJ . Single-cell transcriptomics reveals regulators underlying immune cell diversity and immune subtypes associated with prognosis in nasopharyngeal carcinoma. Cell Res. (2020) 30:1024–42. doi: 10.1038/s41422-020-0374-x. PMID: 32686767 PMC7784929

